# Visual biases in evaluation of speakers’ and singers’ voice type by cis and trans listeners

**DOI:** 10.3389/fpsyg.2023.1046672

**Published:** 2023-05-02

**Authors:** Jay Marchand Knight, Anastasia G. Sares, Mickael L. D. Deroche

**Affiliations:** Laboratory for Hearing and Cognition, Psychology Department, Concordia University, Montreal, QC, Canada

**Keywords:** audio-visual integration, FACH, gender studies, implicit bias, trans voice, voice timbre

## Abstract

**Introduction:**

A singer’s or speaker’s *Fach* (voice type) should be appraised based on acoustic cues characterizing their voice. Instead, in practice, it is often influenced by the individual’s physical appearance. This is especially distressful for transgender people who may be excluded from formal singing because of perceived mismatch between their voice and appearance. To eventually break down these visual biases, we need a better understanding of the conditions under which they occur. Specifically, we hypothesized that trans listeners (not actors) would be better able to resist such biases, relative to cis listeners, precisely because they would be more aware of appearance-voice dissociations.

**Methods:**

In an online study, 85 cisgender and 81 transgender participants were presented with 18 different actors singing or speaking short sentences. These actors covered six voice categories from high/bright (traditionally feminine) to low/dark (traditionally masculine) voices: namely soprano, mezzo-soprano (referred to henceforth as mezzo), contralto (referred to henceforth as alto), tenor, baritone, and bass. Every participant provided voice type ratings for (1) Audio-only (A) stimuli to get an unbiased estimate of a given actor’s voice type, (2) Video-only (V) stimuli to get an estimate of the strength of the bias itself, and (3) combined Audio-Visual (AV) stimuli to see how much visual cues would affect the evaluation of the audio.

**Results:**

Results demonstrated that visual biases are not subtle and hold across the entire scale, shifting voice appraisal by about a third of the distance between adjacent voice types (for example, a third of the bass-to-baritone distance). This shift was 30% smaller for trans than for cis listeners, confirming our main hypothesis. This pattern was largely similar whether actors sung or spoke, though singing overall led to more feminine/high/bright ratings.

**Conclusion:**

This study is one of the first demonstrations that transgender listeners are in fact better judges of a singer’s or speaker’s voice type because they are better able to separate the actors’ voice from their appearance, a finding that opens exciting avenues to fight more generally against implicit (or sometimes explicit) biases in voice appraisal.

## 1. Introduction

In principle, our perception of a speaker’s or singer’s vocal range and *Fach* (or voice category) should rely primarily (if not exclusively) on auditory cues. Yet, in practice, many other cues may be considered, such as the actor’s facial features, height, body size or shape, and potentially, skin color, opening the door to several biases, both unconscious and, in certain circumstances, conscious.

### 1.1. The *Fach* system

*Fach* (the literal translation of which is “compartment” or “subject” but in this context could be taken to mean “specialty”) is a categorization system for voices created in Germany, for the practical purpose of contracting singers for entire opera seasons at a time. In this way, companies could ensure they had the appropriate singers to produce several operas each year and singers/actors could ensure they would not be asked to sing roles that could harm their voices. The system is extremely finicky and now has over 25 categories. These include the large categories of bass, baritone, tenor, alto, mezzo, soprano, as well as the large category of countertenor (generally indicating a cisgender man singing in the treble range and not included in this paper). Within the *Fach* System, there are further divisions of each category into subcategories such as coloratura, lyric, dramatic and more. According to McGinnis, “There are many factors that determine a singer’s *Fach*. These include the singer’s basic vocal equipment, combined with his or her physical appearance, age, and experience” (McGinnis, 2010). Overall range, comfortable *tessitura* (the part of the range in which a singer is most comfortable singing most of the time), size of voice, timbre (often defined as tone colour or sound quality), and level of development are part of *Fach* determination. McGinnis maintains that personality and acting ability play a role in *Fach* determination and, most questionably, physical appearance. She states that large voices do not (or rarely) come in small bodies, etc. These points are important because while an actor may be attracted to dramatic characters, if they do not possess a sizeable voice with a very particular timbre, they would injure themselves singing these roles. However, her theory that big voices only come in big bodies (and conversely small and high voices only come in small bodies) has been subject to criticism (Cotton, 2012).

It is important to mention that all children, prior to the onset of puberty, have vocal apparatuses of similar size and shape. Both testosterone- and estrogen-dominant puberties affect the size of the vocal folds and larynx to different extents: testosterone-dominant puberties generally result in longer and thicker vocal folds as well as a longer vocal tract and bigger larynx than do estrogen-dominant ones. People of all sexes develop a laryngeal prominence (also called Adam’s Apple), as the larynx, which begins as separate, flexible cartilages, descends, fuses, and begins to ossify (Doscher, 1994; Abitbol et al., 1999; Pomfret, 2012; Prakash and Johnny, 2015). The length of the vocal folds contributes to vocal range (frequencies attainable by the singer or speaker), while thickness contributes to vocal weight (how light/lyric or heavy/dramatic the voice is perceived). Vowel formants are partly determined by the length of the vocal tract, with less spacing between formants producing a sound typically associated with cisgender men’s speaking voices (longer vocal tract) and more spaced-out formants producing a sound typically associated with cisgender women’s speaking voices (shorter vocal tract; Sundberg, 1987; Bozeman, 2013; Block et al., 2018; Hirsch et al., 2018).

Exogenous testosterone, often taken by trans masculine people after they have undergone an estrogen-dominant puberty, usually results in some lengthening and/or thickening of the vocal folds (though how much is unpredictable), but because the larynx of an adult has already begun the ossification process, the larynx itself generally will not grow. Likewise, an adult who begins a course of testosterone therapy will generally not get taller or develop a longer vocal tract (Agha and Hynes, 2022). While vocal pitch might change with testosterone therapy alone, many physical elements affecting voice quality will not. Trans women who begin exogenous estrogen therapy will typically not experience any further changes to the voice or vocal anatomy (Gromko and Hatfield, 2018; Loutrari and Georgiadou, 2022). This implies that certain physical attributes are not easily changed by exogenous sex hormone modulations in adulthood and could cue a listener toward features of the voice, in line with McGinnis’s view. Still, other hormones appear to affect the voice: e.g., Rendall et al. (2005) found that higher levels of cortisol, in men, raised their fundamental frequency (F0), even in those with high levels of testosterone.

Other evidence also seems to support the idea that physical factors can inform about features of the voice. For example, neck, waist, chest circumference, weight, or height (but to a smaller degree) might correlate with the F0 of a person’s speaking voice, with larger bodily dimensions predicting lower F0s (Evans et al., 2006; Pawelec et al., 2020). Strikingly, this phenomenon is not only true across individuals of different body types but holds within the same person at different weights: de Souza and dos Santos (2018) focused on obesity in women, finding that after bariatric surgery and weight loss, women’s F0s increased by approximately one semitone and their maximum phonation time increased to match that of a control group of women of normal weight. However, such links (between characteristics of the body and of the voice) are often erroneous. For example, in Van Dommelen and Moxness (1995), listeners did indeed consistently estimate speaker height and weight by relying on the speakers’ F0, vocal tract length/formant spacing, and articulation, but their estimates were not usually correct. Cisgender male listeners (but not cisgender female listeners) tended to accurately judge the height and weight of other cisgender male speakers but neither group of listeners was accurate in their estimates of cisgender female speakers’ bodily dimensions. Similarly, Rendall et al. (2005) found that the relatively large difference in habitual speaking F0 between cisgender men and women did not correlate strictly to differences in body size. As the relationship between body size and F0 seems multifactorial, researchers have looked for other factors that could influence F0 production. Rendall et al. (2005) proposed that vocal fold length was the primary physiological factor behind the large difference in F0 between the speech of cis men and women. Other factors included vocal fold thickness and density, but these have long been contested (Titze, 1989). Finally, they suggested that the adoption of a lower F0 may also be a behavioral choice to advertise a larger body size than the speaker possesses. This latter reason departs from physiology and turns to psychosocial factors. In this vein, Puts et al. (2012) found that lower F0s in men corresponded to increased confidence and aggression, pointing to personality traits. A lack of clear relationship to body dimension is also found for acoustic parameters affecting voice timbre. Height and neck, chest, and/or waist circumference may be a predictor of formant position and spacing (Rendall et al., 2005). Taller (and to a smaller degree, larger) men had darker voices, but the same was not true for women. Ohala (1994) argues that sexual dimorphism in the vocal apparatus occurs in humans around puberty. This manifests in testosterone-dominant puberties as (1) an increase in length and change in texture of the vocal folds and a resultant lowering of the F0 and (2) an increase in the horizontal and vertical dimensions of the vocal tract, resulting in a different formant spacing and sympathetic vibrations, thus producing a rougher vocal timbre. These collectively signal larger size, increased aggression, and greater sexual prowess. While this is seen across species and is likely the aural equivalent of the lion’s mane or the human male’s beard (it makes them seem bigger), Rendall et al. (2005) and Pisanski et al. (2016) claim that men in our culture sometimes create even bigger, darker, lower voices than their anatomy would suggest to signal dominance; similarly, women sometimes raise their F0 and increase formant spacing to appear more youthful, feminine, or submissive, while lowering their pitch when they want appear professional, qualified, and independent (Oakley, 2000; Pisanski et al., 2018). Amazingly, such dimorphism may be seen even in childhood as children as young as 6 are capable of imitating masculine and feminine sounds, despite there being no difference in their vocal anatomy (Cartei et al., 2019). This suggests that the dimorphism we perceive when evaluating gender is in part physical but in part manufactured through cultural habituation.

To summarize, there are physical/biological factors that can influence voice pitch and voice timbre and would support the conception that low/dark voices come in big bodies. Although these findings are interesting, the studies suffer generally from a sample size that is either not sufficiently large or sufficiently diverse to generalize across the entire human population. For example, the study by Pawelec et al. (2020) includes only young men, aged 18–33 from two cities in Poland, showing a lack of diversity; that by Evans et al. (2006) includes only heterosexual, English-speaking men, again showing a lack of diversity; and that by de Souza et al. (2018) involved only 25 morbidly obese participants. Additionally, while there may be average differences in voice types affected by certain biological factors, there is a lot of individual variability, and it should be more important to find a person’s healthy vocal *Fach* than to make them conform to what the average “should” be. There is no need to search for outliers to find famous exceptions to the idea advanced by McGinnis. One need only consider the voices of Luciano Pavarotti, a man of considerable height and weight with a high lyric tenor voice, and Joan Sutherland, a coloratura soprano who sang the highest operatic roles and stood nearly 6 feet tall. The authors would propose instead that society prefers or is accustomed to seeing big and tall actors portray heroic characters and these roles are generally composed for big, dramatic voices. In contrast, small and young-looking actors play romantic leads, and these roles are generally composed for light, high, flexible voices. Very young children have been shown to perceive stereotypical masculinity and femininity in the voice but more fascinatingly, as of the age of 6, children also learn to alter their F0 and vowel formant spacing to masculinize or feminize their voices when imitating stereotypically masculine or feminine characters, although no dimorphic anatomical sex differences in vocal anatomy are present in children prior to age 11. Children have also been shown to make stereotypically masculine or feminine sentence choices based on their audience’s perceived gender (Cartei et al., 2019, 2021a,b). This dependency on the receiver characteristics suggests a particular purpose in modulating one’s voice to match listeners’ expectation of pitch and timbre. Those who have the desired voice-body “match” are the people most often cast, thus reinforcing the erroneous belief that big voices come in big bodies and small voices come in small bodies. This, in effect, is the heart of the bias in voice appraisal which we address in this study.

### 1.2. Implicit and explicit biases in relation to voice

Unconscious or implicit biases may be defined as: “Attitudes and beliefs that occur outside of our conscious awareness and control” (Ruhl, 2020). These can be dissimilar to one’s conscious or explicit biases, meaning one can consciously support the rights of a certain group of people while still holding ingrained, unconscious, or buried biases against that group. It is, therefore, crucial to raise awareness about implicit biases so that we can act to correct our behaviours and be more inclusive (Boscardin, 2015; Staats, 2015; De Houwer, 2019).

Group singing has been shown to have mental health benefits (Clift and Morrison, 2011; Dubinsky et al., 2017; Pullinger, 2020). Unfortunately, transgender people experience exclusion from formal singing due to implicit biases surrounding perceived voice-body mismatches (Janssen, 2018; Eidsheim and Whelden, 2019/2021; Pullinger, 2020) and this also affects cisgender singers whose voices fall outside the traditional *women with high voice and small body* versus *men with low voice and large body* dichotomy. Additionally, there are issues of everyday health and safety for transgender people, who fear outing themselves when speaking in public (Oates and Dacakis, 2015; Jackson Hearns and Kremer, 2018; Mills and Stoneham, 2020; Quinn et al., 2022). Thus, we expect the transgender population to be especially aware of voice-body “mismatches,” and possibly resist them better.

### 1.3. Multimodal basis of these visual biases in voice appraisal

The way the brain perceives gender must integrate different modalities. This multimodal aspect is echoed in the work of Peynircioǧlu et al. (2017), Brang (2019), and Abbatecola et al. (2021), who all designed experiments modeled on either McGurk effect or Ventriloquist effect to investigate auditory–visual interactions in voice/face gender perception. As a reminder, the McGurk effect is an illusion caused by multimodal integration in which the auditory element of one stimulus (e.g., the spoken syllable “ba”) is merged with the visual element of a second stimulus (e.g., the lip movement of “ga”), leading to the perception of an intermediate stimulus (e.g., “da”) which was never presented. The Ventriloquist effect is another illusion in which visual information about the spatial localization of a sound source takes precedence over the spatial cues contained within the auditory information. These illusions can be leveraged to reveal biases in voice appraisal. In the study by Peynircioǧlu et al. (2017), listeners were first presented with audio-only versions of mezzo and baritone singers sustaining a G3 (F0 of 196 Hz). A subgroup of the participants who scored 100% in gender identification of these (purely auditory) voices (noting that authors presumed that an identification of soprano or alto equalled “woman” and an identification of tenor or bass equalled “man”) was then presented with mismatched audio-visual stimuli. For example, a cis woman’s face was paired with a cis man’s voice or a cis man’s face with a cis woman’s voice. The participants’ ability to identify the gender of the singers decreased from 100 to 31%. Despite certain limitations (largely methodological in nature), this study had the merit to demonstrate the strength of visual information even for listeners who were operationalized (by the experimenters’ collapse of the traditional male and female categories into binary distinctions) to appear like they had strict binary views.

Similar cross-modal interactions can be found in the world of music research (Deutsch, 2019). Schutz extensively investigated the role of percussionists’ gestures on listeners’ perception of temporal material (Schutz and Kubovy, 2006, 2009; Schutz and Lipscomb, 2007; Schutz and Manning, 2012). Participants were shown videos of percussionists playing different notes and asked to determine whether the notes were long/sustained or short/staccato. Impressively, all listeners (even the percussionists themselves) could be fooled into believing that notes accompanied by sustained gestures were long, while notes accompanied by the staccato gestures were short, although both videos contained the same audio signal. Note that these cross-modal interactions are not always deceiving. Often, cross-modal interactions exist to support each other. For example, violinists find the sound of their instrument richer when they feel its vibrations as they play than when they hear an audio-only recording of the same note being played (Wollman et al., 2014; Saitis et al., 2017). Singers’ gestures and facial expressions enhanced listeners’ abilities to perceive key changes and emotional meaning in songs (Thompson et al., 2010). In linguistics, cross-modal interactions with facial cues are very common indeed: Strand and Johnson (1996) and Strand (1999) found evidence that the boundary for hearing [ʃ], as in shape (with more energy in the lower part of the spectrum and therefore considered to be a more masculine sound), versus [s], as in sad (with more energy in the higher part of the spectrum and therefore considered to be a more feminine sound) changes for listeners when viewing a masculine or a feminine face speaking.

To summarize, the brain must integrate information from different senses to make a coherent model of the world, but in doing so, it irremediably leads people to make auditory judgments biased by visual cues, and visual judgments biased by auditory cues. In many circumstances, this AV binding is helpful, but it is important to recognize that it is the root cause of the implicit biases aforementioned.

### 1.4. Goal of the present study

If we are to break down biases in voice assessment to create a more equitable environment for speakers and singers regardless of their physical appearance, we first need to explore the conditions under which they occur and provide empirical data to characterize the magnitude and dependency of the biases. On the listener’s side, it could very well be that some individuals are particularly susceptible or particularly resistant to these biases. We reasoned that trans listeners would have some immunity to these biases compared to cis listeners precisely because they are “trained” through their life experience to dissociate voice from appearance. On the singer’s side, it could very well be that altos and tenors suffer less from these biases because they are in the middle of the voice range and so perhaps more versatile (to accommodate for unexpected appearance). Following this reasoning, basses and sopranos might suffer the most from these biases.

To address these research questions, we presented participants with three sets of stimuli of the same 18 actors (who were either speaking or singing) in Audio-only modality (A), Video-only modality (V), and Audio and Visual together (AV) to assess how their ratings of the actor’s voice would change along the gender spectrum after seeing the actor’s face. The 18 actors had vocal types ranging from bass, baritone, tenor, alto, mezzo, or soprano. Participants were taught through a short tutorial what each of these six voice types sounded like (each with spoken and with sung examples). More importantly, we recruited both cisgender and transgender/nonbinary adults. Because transgender and nonbinary listeners are likely to be more aware of the dissociation between a person’s appearance and their vocal characteristics, we hypothesized that these listeners *would resist (in comparison with cis listeners) the influence of visual cues taken from an actor’s face and body type*, and thus provide more consistent ratings across A and AV stimuli.

In addition to this main hypothesis, we explored a few extra parameters, but we did not have strong expectations into their respective roles. (1) We speculated that biases could differ across the human range, and perhaps affect voices on the extremities more strongly (e.g., because the face/body expectations of a bass or a soprano would be further away from the norm). (2) We reasoned that a voice would be easier to judge when pushed outside of its daily use - such as when singing versus speaking - but whether this would lead to stronger weights on auditory versus visual cues (i.e., smaller biases) was largely an open question. (3) Along the same lines, we reasoned that an emotional stimulus (happy or sad utterance) would provide a better glimpse of voice characteristics than a stimulus produced in a neutral manner, but whether this would lead to differential use of auditory and visual cues was again an open question. (4) Finally, we hoped that the semantic content of the sentences being spoken or sung would have negligible effect on the phenomenon observed in this case.

## 2. Methods

### 2.1. Participants

A total of 323 participants were initially recruited through the Prolific (2014) platform, collected in two batches: the first batch recruited was cisgender men and cisgender women (CIS) and the second batch recruited was non-cisgender/gender expansive individuals (TRANS), all between 18 and 31 years of age. Note that within the present TRANS group, there is a diverse community of four sub-groups: transgender men, transgender women, non-binary people assigned male at birth (NB-AMAB), and non-binary people assigned female at birth (NB-AFAB). For clarity, we limited the discussion in the body of the paper to the two umbrella groups of TRANS and CIS.

**Figure 1:**
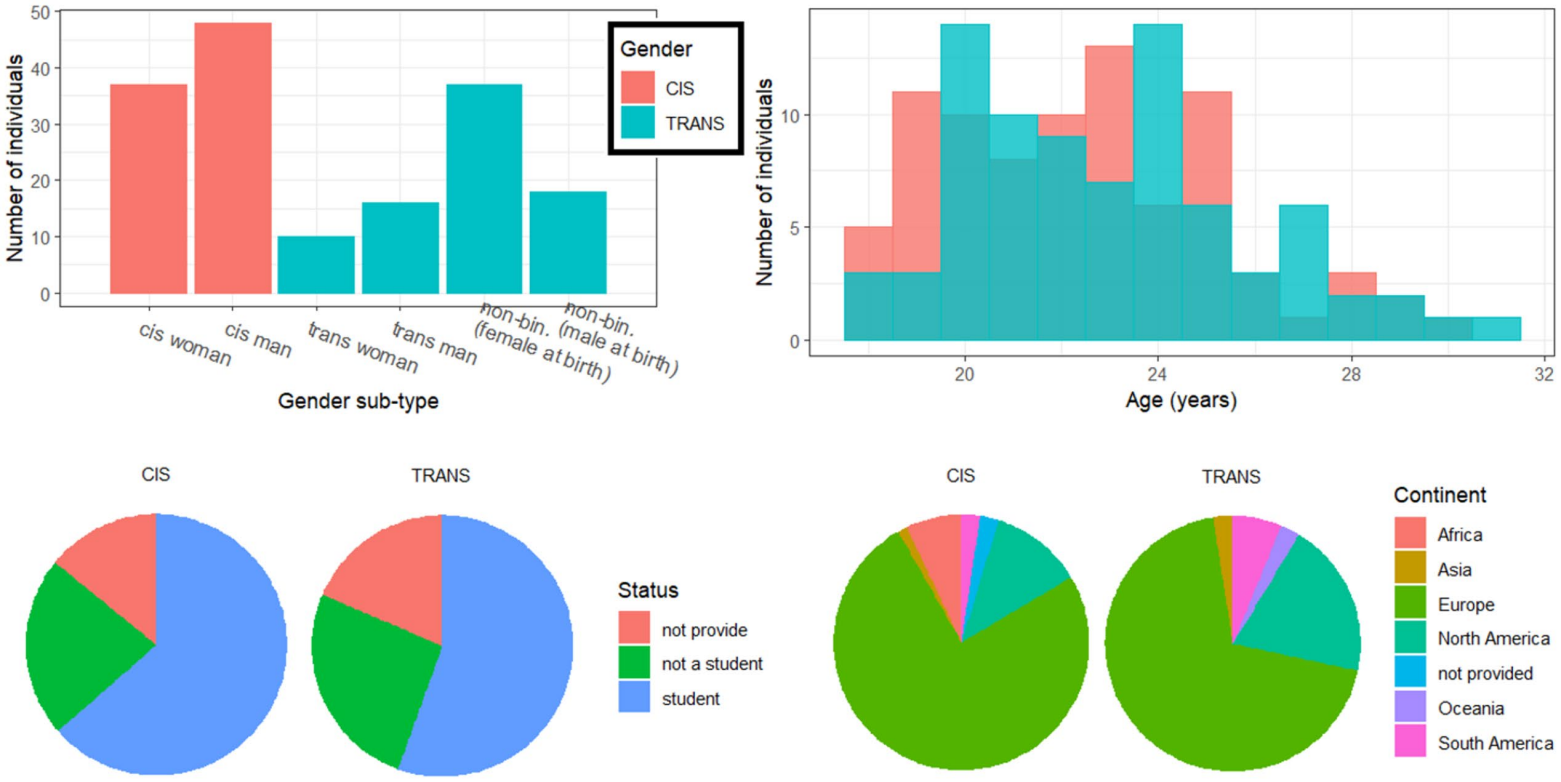
Demographics of the two groups of participants, split by sub-type of gender identity (top left), age at time of testing (top right), student status (bottom left) and geographical origin (bottom right).

A total of 157 had either technical difficulties or did not complete the full study; mostly because the AV materials easily took 15 min to download, which deterred some of these participants before they even began. These (incomplete) files were excluded from any analysis, resulting in 85 participants in the cisgender (CIS) group (37 women and 48 men) and 81 participants in the transgender (TRANS) group (10 trans women, 16 trans men, 37 NB-AFAB individuals, and 18 NB-AMAB individuals). This CIS/TRANS factor was referred to as *gender* (not sex). Additional analyses on the sub-types within the TRANS group can be found in Supplementary material 2, along with male vs. female assigned sex differences in Supplementary material 3. As illustrated in the top-right panel of Figure 1, the two groups were not statistically different in age (about 22.7 +/− 2.9 years) [t(163) = −1.3, *p* = 0.211], in student status [χ^2^(2) = 1.2, *p* = 0.561, about 60% students], employment status [χ^2^(6) = 5.5, *p* = 0.486], geographical origin [χ^2^(2) = 2.0, *p* = 0.364], and took a similar amount of time to complete the study (about 85 +/− 22 min) [*t*(163) = 0.6, *p* = 0.549]. Arguably, this is fairly long and fatigue could have set in toward the end of the experiment but it affected all conditions equally (due to randomization of stimuli) and would be difficult to measure (given that there was no technically correct or incorrect response). To summarize, except for their gender identity, the two groups were matched in most variables to which we had access from their Prolific profiles.

### 2.2. Stimuli

Stimuli were selected from the Ryerson Audio-Visual Database of Emotional Speech and Song (RAVDESS) (Livingstone and Russo, 2018). The RAVDESS is comprised of 7,356 files of 24 professional actors singing and speaking two phrases: “dogs are sitting by the door” and “kids are talking by the door.” The emotions portrayed in the speech samples are happy, calm, sad, angry, fearful, surprised, and disgusted. Emotions portrayed in the sung samples are happy, calm, sad, angry, and fearful. Phrases were delivered at normal and strong levels of emotional intensity. Each sample exists in a V version (no sound), an A version (16-bit, 48 kHz), and a combined AV version (720p H.264, AAC 48 kHz, .mp4).^1^

Of the 24 actors in the RAVDESS corpus, 18 were chosen for inclusion in the study because their voices were judged to fit within the six large categories of the German *Fach* System of voice classification. This evaluation was done by first author JMK, relying on their extensive background in opera performance and vocal pedagogy as well as their training in timbre studies. The six categories are (from low/dark to high/bright): bass, baritone, tenor, alto, mezzo, soprano. Traditionally, (cisgender) women have been placed in the three highest categories while (cisgender) men have typically been placed in the three lower categories. Note that for this study, the category of countertenor voice, or (cisgender) male treble, was not included because no countertenor voices were represented in the RAVDESS corpus.

Uncomfortable as it may be, we also sought to investigate whether certain actors (because of their appearance) would be more affected by listener bias than others, depending on whether they were a visual match for their *Fach*. The determination of voice-body match or mismatch was made based on JMK’s knowledge and experience with casting trends using heuristics rules that are questionable but often relied upon. No objective validation of this classification is available (nor was it critical for our primary aim) but sources such as blogs and magazine articles depict examples of voice-body mismatch that we encountered and that continue to be problematic for auditioning opera singers (McNeil, 2015; Salsbery Fry, 2018; By Voice Alone, 2019; Hahn, 2021). Three actors were chosen in each category, whose face/body size and shape were presumed to indicate an upward mismatch (i.e., whose voice sounded lower/darker than their physical appearance suggested), a downward mismatch (i.e., whose voice sounded higher/brighter than their physical appearance suggested), or a match. This factor was referred to as *appearance direction.*

Although voice emotion was not the focus of the present study, we reasoned that a voice might perhaps be better evaluated when pushed to its extreme, i.e., when the actor was instructed to sing rather speak, and when instructed to enact a high-intensity emotion rather than a neutral emotion. These two variables were designed in an orthogonal manner such that there was an equal number of spoken and sung stimuli (referred to as *mode*), and within each mode there was an equal number of neutral and emotional stimuli (referred to as *emotional intensity*). Note that only the happy or sad versions of the recordings were chosen for the emotional condition, and specifically those enacted with high emotional intensity in an attempt to enhance the emotional contrast. Finally, there was an equal representation of the two sentences, in each combination of emotional intensity and mode. This resulted in 18 actors × 2 modes × 2 emotional intensities × 2 items = 144 AV recordings. Additional analyses are described in Supplementary material 1 to demonstrate that neither the emotional content nor the semantic content had much impact on the key findings of this study.

### 2.3. Design and procedure

Prior to beginning the tutorial and the experiment, all participants answered demographic questions about their ages and gender identities (note that some information on gender identity is collected by Prolific but we wanted participants to self-identify in the moment as gender identities can be subtle and can shift over time). Then, participants answered a 20-question survey on gender views (see Supplementary material 5; Tezare, 2015).

Participants first completed a short tutorial in which they were exposed a single time to the 6 voice types in the study (bass, baritone, tenor, alto, mezzo, soprano) with A-only stimuli (same actors but different samples from those used in the test, both spoken and sung). They were told the correct category and instructed how to move the slider along the line, representing a spectrum from low/dark (bass) to high/bright (soprano) voices with labels underneath. By showing the voice types on a continuous spectrum, we hoped to discourage participants from making binary judgments about the speaker’s or singer’s gender and encourage them to think of voices in terms of pitch/timbre continuums.

Having completed this tutorial (where no subject was ever excluded based on their performance in this tutorial because the task was not intended as a test), participants proceeded to the experiment. In phase 1 of the study, participants judged all 144 A-only stimuli by moving a slider along the low/dark-high/bright continuum. In phase 2 of the study, participants were asked to again move the slider but this time they viewed silent videos of the same recordings they had rated in phase 1 (V-only). Finally, in phase 3 of the study, participants were presented with the full AV versions of the same 18 actors and reported their voice appraisal on the same low/dark-high/bright continuum. Critically, our analysis focused on comparing ratings between modalities, and as such there was no correct or incorrect response. Our goal was not to measure how accurate participants were at allocating a voice type to a given actor but to assess how their ratings would change once they saw the actor. The order of the 144 stimuli was shuffled randomly for each block and each participant, but the order of blocks was always A, then V, then AV. This was meant to prevent the possibility of pairing the audio with visual information on a given actor while still completing single-modality ratings (which would arguably have reduced the bias we intended to observe). Before exiting the online interface, participants were asked to provide feedback on five questions pertaining to possible technical difficulties, the amount of mental effort in completing the task, clarity of the instructions and extent of the practice, goal of the study, and whether they noticed anything special with the stimuli. No data from these questions is reported here because they did not raise any finding or concerns. All participants provided informed consent in accordance with the Institutional Review Board at Concordia University and were compensated $10 CAD for their online participation.

### 2.4. Equipment

This experiment was run fully online, with no direct control over participants’ audio equipment. There was no difference between CIS and TRANS in the type of audio device used [χ^2^(3, *N* = 166) = 6.1, *p* = 0.107]: about 45% of participants used headphones, 17% used external speakers, 16% used earbuds, and 22% used their default laptop output. Participants were instructed to set their sound at a comfortable level during practice and not change it afterwards. They indicated the quality of their audio from 1 (poor) to 5 (excellent), and the two groups did not differ in this regard [*F*(1, 164) = 2.3, *p* = 0.135] with a mean (sd) of 4.2 (0.7).

## 3. Data analysis

### 3.1. Gender views

For each question in the survey on gender views, there were 4 possible choices: 1 for “completely disagree,” 2 for “somewhat disagree,” 3 for “somewhat agree,” and 4 for “completely agree.” These ordinal data were examined with a generalized linear model using a binomial distribution.

### 3.2. Ratings for A-only and V-only stimuli

Before addressing biases in AV material, it was necessary to examine the pattern of responses in each modality, respectively. A repeated-measures analysis of variance (rm-ANOVA) was conducted on the A-only and V-only ratings with one between-subject factor: gender of the listener (cis vs. trans), and two within-subject factors (voice category of the stimulus with 6 levels, namely bass, baritone, tenor, alto, mezzo, soprano, and mode of production with 2 levels namely spoken vs. sung). Degrees of freedom were corrected with Greenhouse–Geisser adjustments which were necessary for the effects that involved voice category, as the assumption of sphericity was always violated. Also, homogeneity of variance between the two groups was respected overall but this was not necessarily true on a voice category × mode condition. Thus, we recruited bootstrapping techniques to corroborate our findings without the need for these assumptions; this provided more reliable estimates of means and confidence intervals and all *p*-values were in good agreement with traditional statistics (see Supplementary material 4 for further details).

### 3.3. AV shifts in the direction of the visual bias

**Figure 2:**
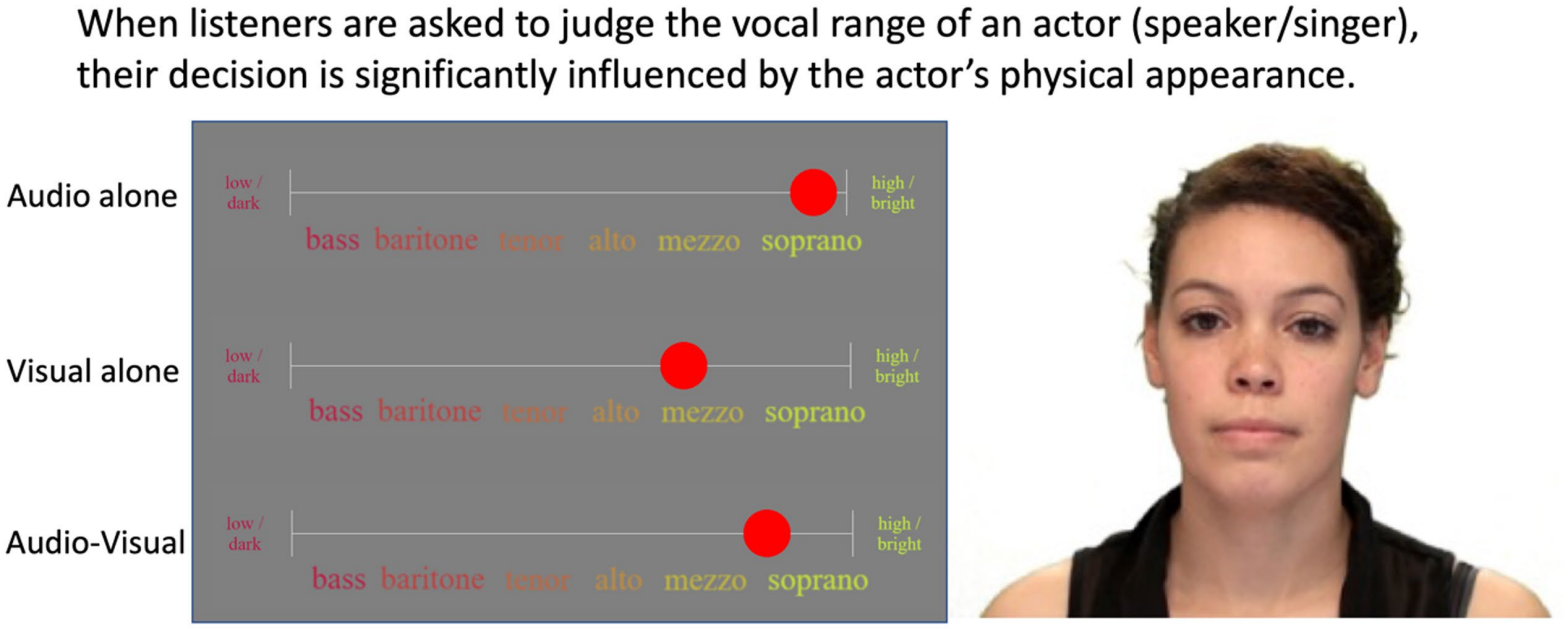
Design and interface showing a representative set of A/V/AV ratings for a single actor (not the task screen). Photo is available as part of the RAVDESS database and is reproduced with permission from Steven R. Livingstone and Frank A. Russo (Livingstone and Russo, 2018).

The key analysis of this study was to determine whether participants were influenced by the visual cues (the actors’ appearance and mannerisms) *when explicitly asked to rate the voice* in the AV stimuli. In other words, we were interested in the AV – A difference, and specifically whether it went in the direction of the V – A difference. As an example, a participant could give a rating of 5.8 when listening to the soprano actor in Figure 2, but when seeing the actor in question, they might give a rating of 4.8 (because their appearance would somehow suggest a darker/lower voice). When watching the full AV material, this participant might respond somewhere between their A and V ratings, perhaps giving a rating of 5.6. Thus, compared to their original A rating, they would have shifted by 0.2 in the direction suggested by the visual cues. This shift was the key dependent variable of this study.

One might intuitively think that the larger the mismatch between A and V ratings, the larger the AV shift might be, and thus one might wish to express it as a percentage of the V shift. Unfortunately, as this analysis was done for every single item in a paired fashion, there were many instances where the A and V rating could be very close to one another (and in fact sometimes identical), leading to disproportionately large proportional AV shifts (sometimes infinite). Instead, we opted to use the raw values, with the only manipulation being to multiply the (AV – A) difference by the sign of the (V – A) difference, to standardize the AV shift in a consistent direction (i.e., toward the visual bias – for positive values - or away from the visual bias – for negative values). As with single modality ratings, a rm-ANOVA corroborated by bootstrapped estimates of the important means and their 95% confidence intervals was conducted on the (signed) AV-A shift, with the same three factors (gender, voice category, and mode). Note that we also analyzed the V – A metric and the results were consistent with findings observed in each modality, so we did not report it here to avoid redundancy.

Two additional analyses were conducted on the (signed) AV-A shifts. The first focused on their correlation with the participants’ gender views. Linear regressions were performed between each person’s average score from the gender views questionnaire and their average AV shift. This was done for each of the six categories separately. The second additional analysis attempted to split the AV shifts going upwards versus those going downwards to examine whether there could be some directionality effect in the size of the bias observed. To this aim, we replaced mode by appearance direction in the rm-ANOVA.

## 4. Results

### 4.1. Gender views

**Figure 3:**
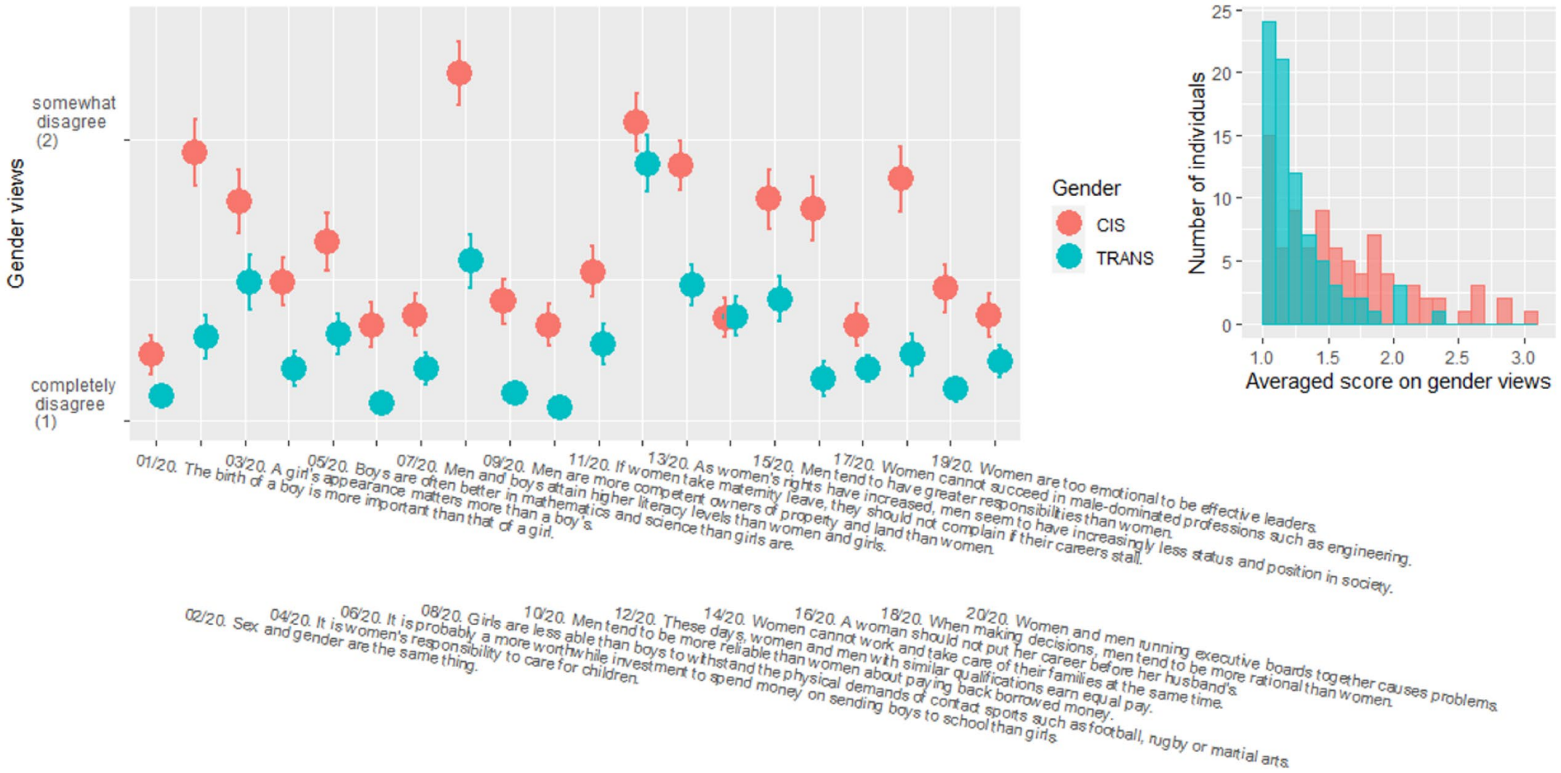
Scores obtained by cis (red) and trans (blue) participants in the questionnaire evaluating gender views, with means and standard errors in each population (left panel). Histogram of the averaged score across the 20 questions in each population (right panel). See Supplementary material 5 for a full list of the gender views questions.

Figure 3 shows the responses to the questionnaire on gender views, with each question labelled. There was a main effect of gender [χ^2^(1) = 25.0, *p* < 0.001], with trans participants holding more progressive views on gender than cis participants. There was also a main effect of questions [χ^2^(19) = 777.0, *p* < 0.001], as agreement differed across the different topics covered in the questionnaire. And there was a significant interaction between gender and questions [χ^2^(19) = 116.4, *p* < 0.001] because the effect of gender was significant in 14 out of the 20 questions (*p* < 0.010). Note that within the six questions that did not show significant differences, question 14 (“women cannot work and take care of their families at the same time”) was really the only one with no sign of a group difference; the others were at least qualitatively going in the direction of the main effect. To summarize, TRANS participants held more progressive views overall on gender than CIS participants, and this was more apparent in some questions than in others.

### 4.2. Ratings for A or V alone

**Figure 4:**
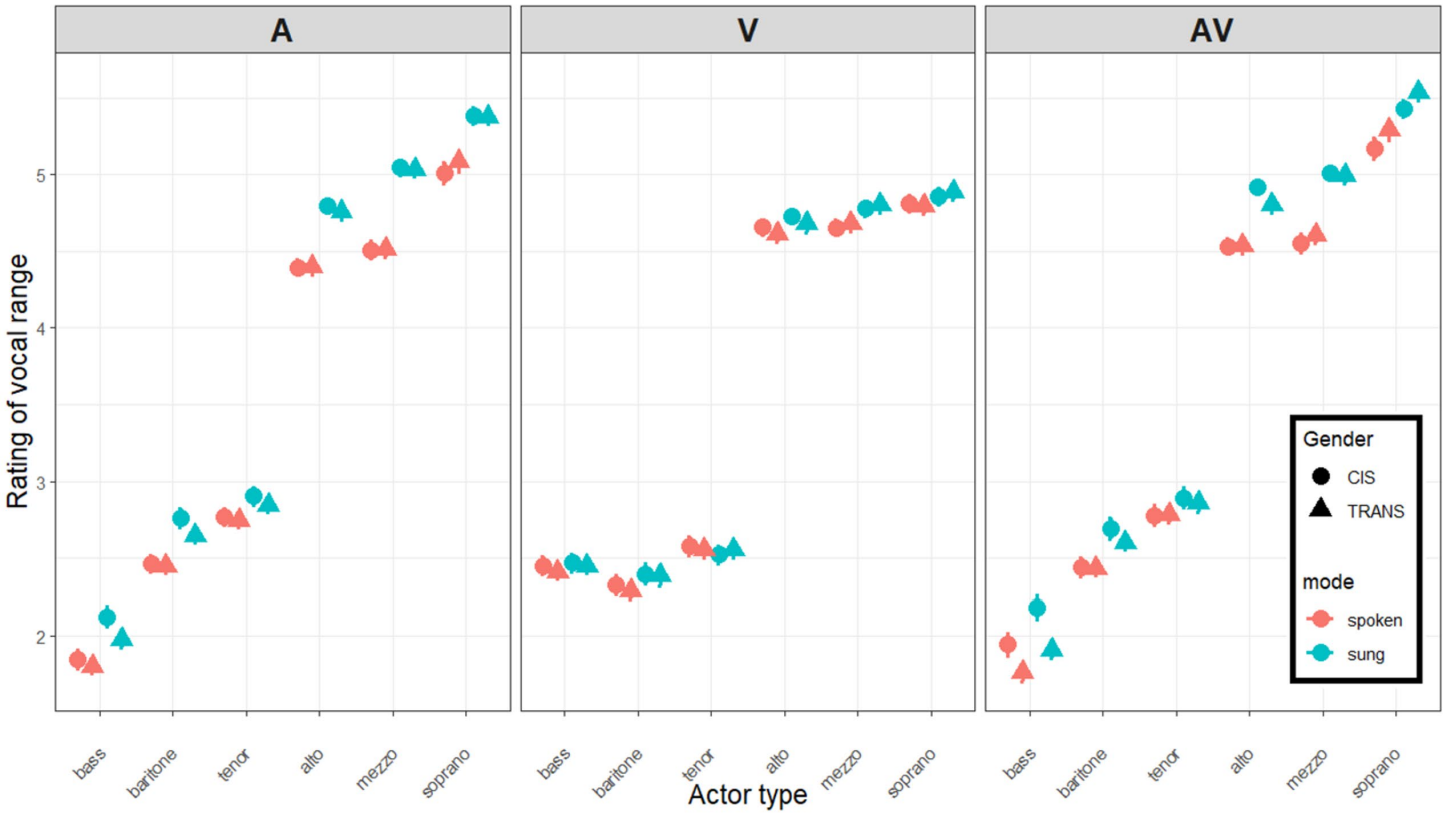
Mean ratings of six categories of actors (singers in blue; speakers in red), conducted by cis (circles) and trans (triangles) participants listening to A stimuli alone (left panel), watching the V silently (middle panel), or with both A and V on (right panel). Error bars show one standard error from the mean.

Based on the instructions during the tutorial phase, ratings were expected to be around 6 for sopranos, 5 for mezzos, 4 for altos, 3 for tenors, 2 for baritones, and 1 for basses. As illustrated in Figure 4, listeners did not follow this 6-point scale and instead compressed it roughly to a 3.5-point scale. This is common as participants are often reluctant to respond toward the extremities of any given scale, while still learning about the diversity of experimental stimuli. This was just a scaling issue and posed no constraint to our analysis as we were mostly interested in relative changes from A to V and eventually to AV stimuli, but it is worth bearing in mind that the magnitude of these shifts depended to a small degree on the degree of compression exhibited by a given participant. For the sake of transparency, however, we decided not to normalize the individual participant scales in any way.

*A-only ratings:* As expected, there was a main effect of voice category [*F*(1.5,239.1) = 1574.3, *p* < 0.001, η^2^ = 0.851] reflecting that listeners were (to some degree) capable of allocating actors to each category in the correct order. Bootstrapped estimates were 1.932 [1.869–2.019] for bass, 2.579 [2.520–2.653] for baritone, 2.814 [2.754–2.885] for tenor, 4.589 [4.527–4.652] for alto, 4.778 [4.711–4.838] for mezzo, and 5.220 [5.131–5.292] for soprano. A gap is notable between the bottom three (traditionally male) and the top three (traditionally female) categories. There was a main effect of mode [*F*(1,164) = 266.3, *p* < 0.001, η^2^ = 0.013] reflecting higher ratings in sung than in spoken stimuli. Bootstrapped estimates were 3.499 [3.460–3.537] for spoken stimuli and 3.806 [3.769–3.847] for sung stimuli. Furthermore, this mode difference (+0.307 point) was more pronounced for some categories, e.g., mezzos (+0.229, +0.248, +0.115, +0.382, +0.528, +0.333, respectively, in bass, baritone, tenor, alto, mezzo, soprano), resulting in an interaction between mode and category [*F*(4.1,680.1) = 34.3, *p* < 0.001, η^2^ = 0.002]. Gender (i.e., CIS vs. TRANS listeners) did not result in a main effect [*F*(1,164) = 0.7, *p* = 0.391, η^2^ < 0.001], and did not interact with voice category [F(1.5,239.1) = 0.4, *p* = 0.579, η^2^ < 0.001], with mode [*F*(1,164) = 2.8, *p* = 0.097, η^2^ < 0.001], or in a 3-way [*F*(4.1,680.1) = 0.5, *p* = 0.735, η^2^ < 0.001].

To summarize, data from the first phase demonstrated that participants were able to categorize voices successfully, illustrated by the diagonality of ratings in the left panel of Figure 4. Sung stimuli received higher ratings than spoken stimuli (perhaps because they are more expressive and that these qualities tend to be more strongly associated with feminine traits; McConnell-Ginet, 1978; Brody, 1993; Briton and Hall, 1995; Vasyura, 2008; Jackson Hearns, 2018). Trans listeners did not differ from cis listeners in their rating of this single modality.

*V-only ratings:* As expected, there was a main effect of voice category [*F*(1.5,250.1) = 1206.0, *p* < 0.001, η^2^ = 0.842] reflecting that participants made a guess on the actors’ voice type (despite the videos being silent). Ratings globally fell into traditional male vs. female categories: bootstrapped estimates were 2.442 [2.378–2.532] for bass, 2.345 [2.272–2.440] for baritone, 2.549 [2.480–2.643] for tenor, 4.674 [4.586–4.734] for alto, 4.730 [4.654–4.791] for mezzo, and 4.842 [4.750–4.906] for soprano. Clearly, participants relied heavily on a binary allocation from the actors’ face and body size/shape. Unexpectedly, the main effect of mode was again significant [*F*(1,164) = 26.1, *p* < 0.001, η^2^ < 0.001] reflecting higher ratings in sung than in spoken stimuli. Bootstrapped estimates were 3.568 [3.532–3.611] for spoken stimuli, and 3.625 [3.593–3.670] for sung stimuli. This mode difference (of +0.057 points) was more pronounced for baritones and mezzos than other categories (respectively +0.029, +0.085, −0.028, +0.066, +0.123, +0.069 for bass, baritone, tenor, alto, mezzo, soprano), resulting in an interaction between mode and category [*F*(4.4,719.5) = 7.4, *p* < 0.001, η^2^ < 0.001]. Gender did not result in a main effect [*F*(1,164) < 0.1, *p* = 0.797, η^2^ < 0.001], and did not interact with voice category [*F*(1.5,250.1) = 0.1, *p* = 0.813, η^2^ < 0.001], with mode [*F*(1,164) = 1.0, *p* = 0.309, η^2^ < 0.001], or in a 3-way [*F*(4.4,719.5) = 0.4, *p* = 0.826, η^2^ < 0.001].

To summarize, as illustrated in the middle panel of Figure 4, the silent videos instilled in all participants a *binary* sense of the actors’ vocal potential (for lack of a better word), with a very slight tendency for some actors to appear higher/brighter when it looked like they were singing as opposed to speaking (perhaps for the same reason as aforementioned, namely that faces often seem more expressive when singing, and emotional expressivity tends to be associated with more feminine traits). Trans listeners did not differ from cis listeners in their rating of this single modality.

### 4.3. AV shifts in the direction of the visual bias

**Figure 5:**
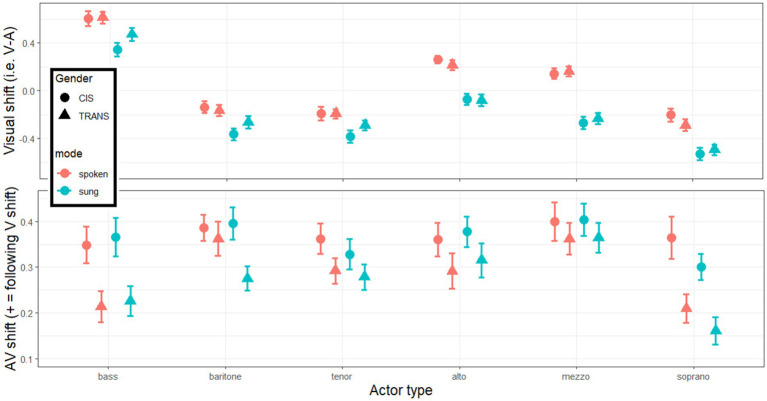
Visual shifts (top panels) calculated from the difference V – A ratings in each category of actors, each mode, and each group of listeners. AV shifts (bottom panels) calculated from the difference AV – A ratings multiplied by the sign of V shift so that positive value always indicate a shift toward the visual influence.

Before analyzing the AV shifts, and specifically whether they followed the direction of the visual biases, it is useful to qualitatively describe the pattern of these visual shifts, i.e., V-A differences. Since V-only ratings were largely flat across the bottom three categories (traditionally male) and the top three categories (traditionally female) with a gap in between, while A-only ratings increased more incrementally with category, this resulted in V shifts exhibiting two downward diagonal patterns across the three bottom and three top categories, respectively (Figure 5, top panels). Also, the effect of mode was substantially stronger in A-only than in V-only stimuli, which translated to the V shifts being overall lower or more negative for sung than for spoken stimuli. These visual shifts were analyzed carefully, but they were consistent with the observations discussed earlier and will not be repeated here. The key metric extracted from these V shifts was their direction. When the sign of the V-A difference was positive, it meant the actors’ appearance suggested a brighter or higher voice than the actor actually had. When the sign of the V-A difference was negative, it meant that the actors’ appearance suggested a darker or lower voice than the actor actually had.

With this in mind, we can now turn to testing the main hypothesis related to AV ratings. First, simple t-tests revealed that the AV shift was significantly above 0 in each category and each population (*p* < 0.001 in all 12 cases, including Bonferroni corrections), demonstrating that AV ratings were *systematically biased* toward the direction of the visual cues. This is one key finding here, as it is one of the first studies to capture the magnitude of this phenomenon with a decent granularity. Furthermore, as illustrated in the bottom panels of Figure 5, there was a main effect of gender [*F*(1,164) = 7.3, *p* = 0.007, η^2^ = 0.018] reflecting that *the AV shift was 30% larger for CIS than for TRANS listeners.* On average across actor categories, bootstrapped estimates were 0.366 [0.327–0.429] for CIS participants and 0.279 [0.246–0.331] for TRANS participants. This is a second key finding of the study, supporting our hypothesis. There was also a main effect of category [*F*(4.4,727.8) = 9.2, *p* < 0.001, η^2^ = 0.016] as the AV shift was reduced for basses and sopranos (bootstrapped estimates of, respectively, 0.289 [0.246–0.342] and 0.259 [0.222–0.305]) relative to actors in the middle of scale (bootstrapped estimates of, respectively, 0.356 [0.323–0.397], 0.316 [0.282–0.355], 0.336 [0.300–0.387], and 0.383 [0.346–0.430] for baritones, tenors, altos, and mezzos). Interestingly, the interaction between voice category and gender approached significance [*F*(4.4,727.8) = 2.2, *p* = 0.056, η^2^ = 0.004] and would have suggested that the resistance of TRANS listeners occurred more toward actors on the extremities of the human range such as basses and sopranos than toward categories in the middle. Finally, there was no main effect of mode [*F*(1,164) = 1.2, *p* = 0.271, η^2^ < 0.001], or mode by category interaction [*F*(4.6,754.3) = 1.5, *p* = 0.178, η^2^ = 0.002], or mode by gender interaction [*F*(1,164) = 0.2, *p* = 0.666, η^2^ < 0.001] or a 3-way interaction [*F*(4.6,754.3) = 0.7, *p* = 0.583, η^2^ < 0.001]. In other words, the bias observed was identical whether actors spoke or sung, and this was true regardless of the actor or the listener. This is not necessarily intuitive, considering that mode has a systematic effect in single modalities (sung stimuli being rated as higher/brighter) but since it applies to A, V, and AV, it has negligible impact on this metric. Note that the same is true of the impact of emotional content (see Supplementary material 1).

### 4.4. No relationship to gender views

**Figure 6:**
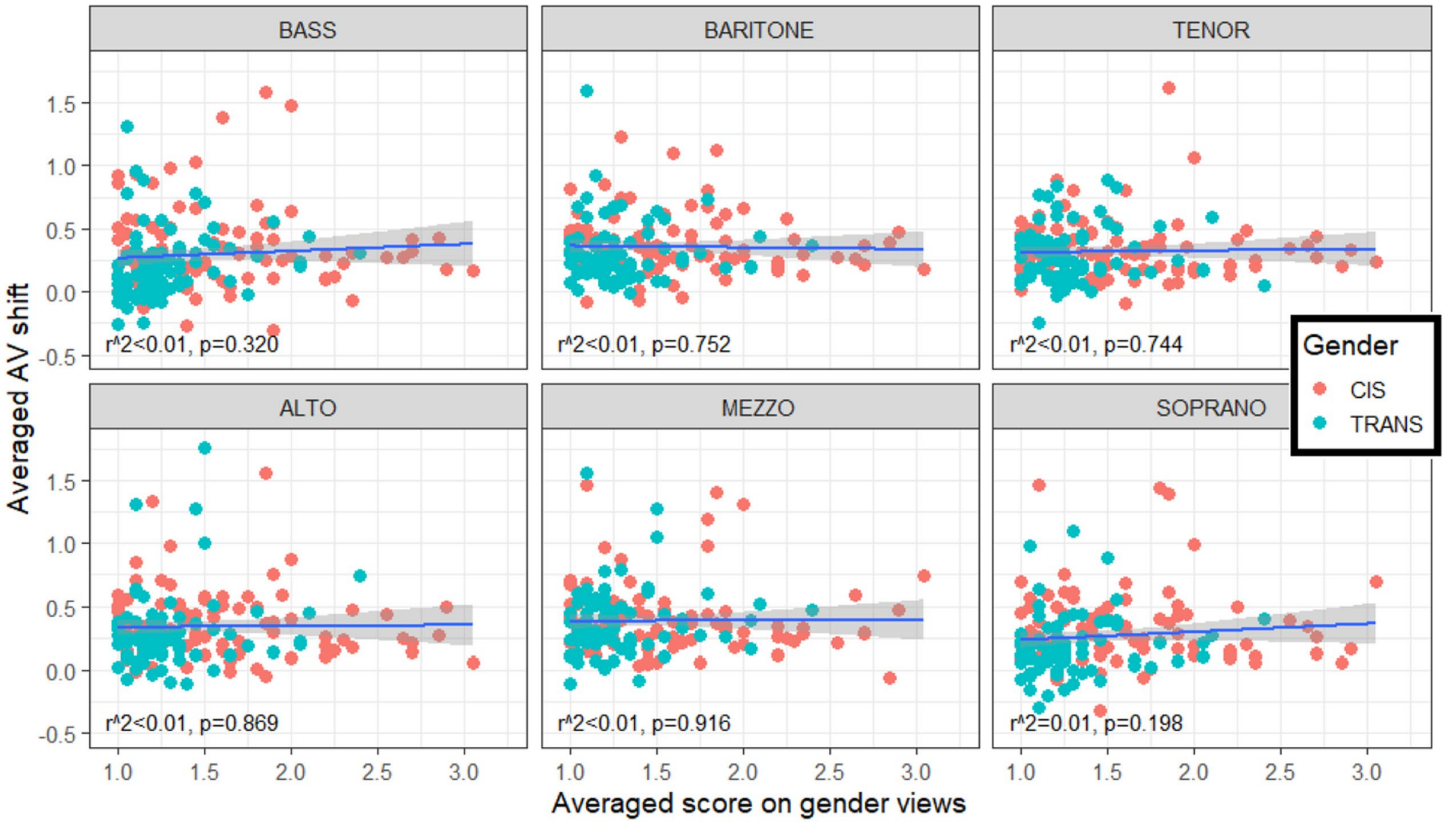
Lack of correlation between individuals’ average score on gender views (abscissa) and their average AV shift (signed, so that positive values indicate a shift in the direction of the visual bias). The degree to which a given participant was biased in their appreciation of vocal range by the visual cues taken from the actors’ face/body size and shape was not related to their views of gender roles in society.

Since TRANS participants held more progressive views on gender (section 4.1) and demonstrated an enhanced ability to ignore visual biases when making vocal judgments (section 4.3), we were curious to probe whether a link existed between the two. Linear regressions were conducted between the AV shift (still signed, so that positive values varied with the V shift direction) of each participant with their averaged score on gender views, and this was done for each category separately. Figure 6 illustrates that none of the regressions were significant (*p* > 0.198, and neither were there significant correlations within trans participants only, or within cis participants only). This lack of correlation implies that gender views (which are largely opinions of gender roles in society) are presumably not the underlying mechanism that gives trans participants an advantage in resisting visual biases in this task. We surmise that it is specifically the life experiences of trans individuals and the mental dissociation between physical appearance and voice quality appraisal that are at the root of this benefit.

### 4.5. Direction of the match/mismatch with actors’ physical appearance

**Figure 7:**
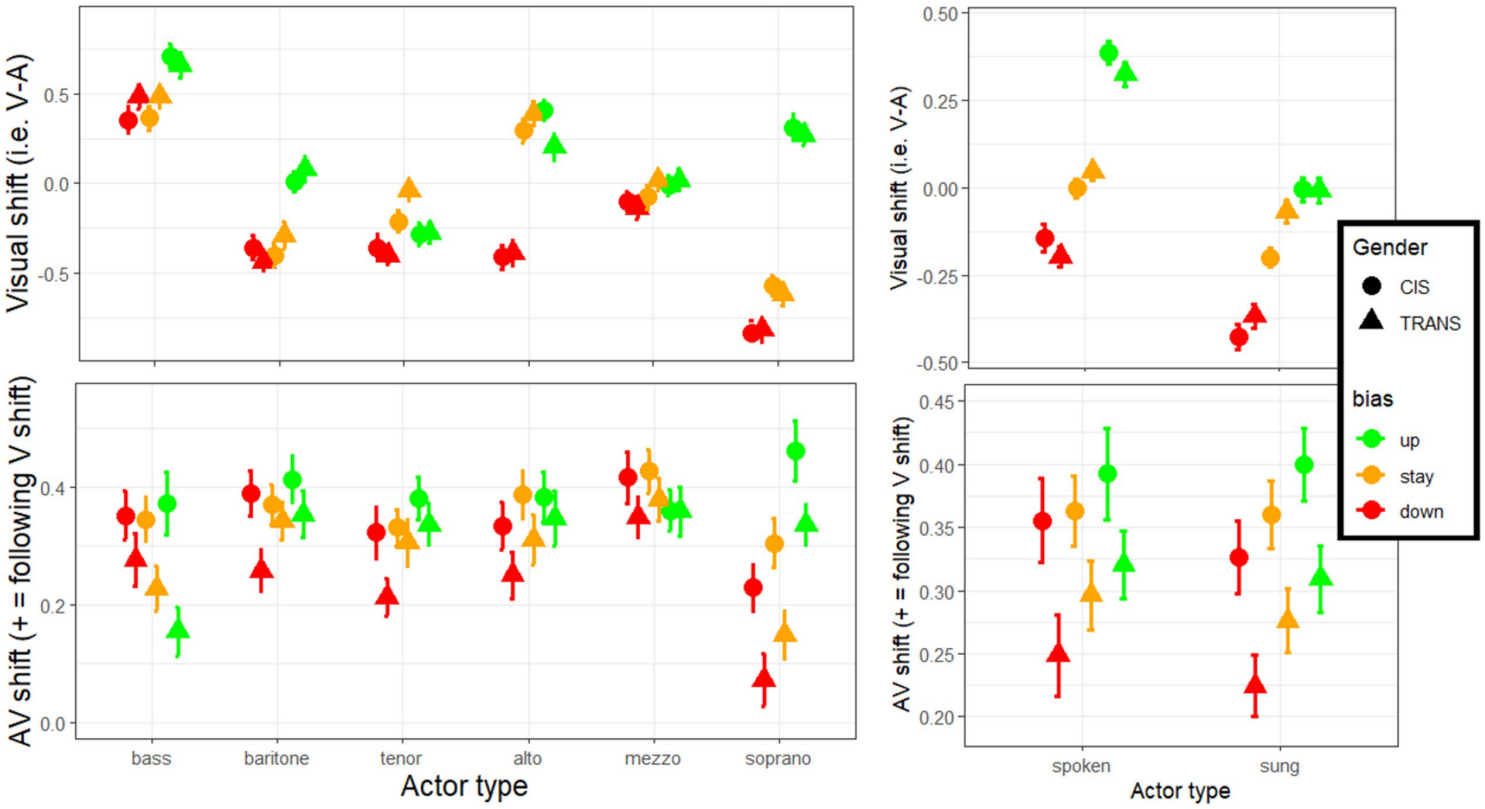
Size of the V shift and AV shift, split by the actor’s match or mismatch between their appearance and their voice.

In the analyses described above, the AV shift was always expressed as a positive value when it followed the direction of the visual bias. AV ratings (shown in Figure 4, right panel) did not appear massively different from A ratings, despite being systematically 0.2–0.4 point shifted in the V direction, because these upward and downward effects counteracted each other. Remember, however, that three actors were chosen in each category to be upwards misfits (referred to as up-actors), downwards misfits (referred to as down-actors), or relatively good fits (referred to as stay-actors) based on heuristics commonly used in casting trends. Although we would generally object to such heuristics, we thought it was an interesting exercise to contrast upward vs. downward biases. An analysis was conducted as in section 4.3 but adding *appearance direction* as an additional factor. This new analysis revealed a main effect of appearance direction [*F*(2,328) = 11.8, *p* < 0.001, η^2^ = 0.005], reflecting that the magnitude of the AV shift was greatest with up-actors (0.36), intermediate with stay-actors (0.32) and weakest with down-actors (0.29), with each pairwise comparison significant [*p* < 0.023]. This is best illustrated in the bottom-right panel of Figure 7 (similarly whether actors spoke or sung). However, appearance direction interacted with category [*F*(9.1,1486.3) = 5.7, *p* < 0.001, η^2^ = 0.012]: as illustrated in the bottom-left panel of Figure 7, it had little/no role for basses (*p* = 0.328), baritones (*p* = 0.142), altos (*p* = 0.089), and mezzos (*p* = 0.313); but some role with tenors (*p* = 0.017) and sopranos (*p* < 0.001), where a greater AV shift occurred toward up-actors than down-actors. Importantly though, appearance direction did not interact with gender [*F*(2,328) = 0.6, *p* = 0.541, η^2^ < 0.001] or in a 3-way interaction [*F*(9.1,1486.3) = 1.0, *p* = 0.424, η^2^ = 0.002]. A rather unexpected trend is notable among basses where TRANS participants experienced the effect of appearance direction in the opposite direction to its main effect (i.e., greater AV shift with down-actors than with up-actors) which suggests that the resistance of TRANS participants in basses is particularly induced by actors whose appearance reflects a higher voice type than they actually had. But the lack of 3-way interaction should prevent us from commenting on this any further: as a first approximation, the two groups did not differ in their dependency to appearance direction, regardless of actor category. In a different analysis, we also replaced voice category by mode, but appearance direction did not interact with mode [*F*(2,328) = 0.5, *p* = 0.635, η^2^ < 0.001] or in a 3-way interaction (with mode and gender) [*F*(2,328) = 0.1, *p* = 0.877, η^2^ < 0.001].

Overall, this analysis indicated that we were successful (to some degree) in generating biases in both upward and downward directions. As we had intended, the visual biases tended to be positive for up-actors, mixed for stay-actors, and negative for down-actors (top-right, Figure 7). But it is interesting to observe that the AV shifts were largest with up-actors and lowest with down-actors, inferring some degree of directionality to the phenomenon discussed in this study: somehow the actors who suffer from the most inaccurate judgment of their voice are those who look more feminine than their voice reveals; actors who look more masculine than their voice reveals are relatively more spared. This directionality effect appears applicable to trans participants as much as to cis participants, and regardless of whether actors spoke or sang.

## 5. Discussion

Overall, the current study shows that a person’s impression of vocal type is pulled toward a gender binary when visual cues are present, while their reliance on auditory cues would allow for a more nuanced, graded view. Importantly, this influence of the visual binary affects cisgender observers more than transgender observers. Such misclassifications of voice type can negatively impact singers’ health.

With only a short tutorial on what basses, baritones, tenors, altos, mezzos, and sopranos sounded like, listeners were capable of roughly placing the voice of different actors (here 18) at different points on a continuous scale from low/dark to high/bright. With visual-only stimuli, however, participants largely fall back on a dichotomous allocation. There is a lot of information within these silent videos (body type, facial expression, breathing dynamics, articulatory motion), but our findings imply that people make little use of these potentially subtle visual cues when rating *Fach*. In other words, visual perception of speakers and singers remains binarily divided along traditional gender lines of man/woman, compared to its auditory counterpart. With AV stimuli, participants could have relied almost exclusively on powerful auditory cues, which led to more gradual categorization, rather than on visual cues which led to poor discrimination. Yet this is not what they did; all participants were pulled toward a gender binary to some degree by visual information.

The size of the effect found here was on average 0.2 to 0.4 points on a 6-point scale across all actors. This phenomenon is a perfect example of visual dominance (Colavita, 1974) despite its apparent ineffectiveness in categorizing voices, but this dominance might not be set in stone, as trans listeners were here able to resist it, at least to some degree. This is perhaps the most exciting finding of this enterprise, suggesting that trans individuals are better candidates than most, for objectively evaluating the true size, timbre, range/*tessitura* of voice *Fach* as well as the gender cues present in natural speech. In fact, we found this trans advantage to be slightly amplified for actors on the extremities of the scale (i.e., basses and sopranos). Unfortunately, despite this trans advantage for judging voices of all types, it is cis listeners who are usually called upon to judge trans voices, in both musical and non-musical/speech related contexts. Here, we will first review the relevant literature on AV integration and discuss the extent to which one could act against it to limit the current biases in voice appraisal. Second, we will discuss the literature more applicable to the trans community.

### 5.1. Audio-visual integration, an obligatory process?

AV integration appears to be obligatory to a certain extent and useful in most contexts, as it can help us decipher the world around us (e.g., lip reading, identifying a speaker’s mood, to support communication). However, this means that in the presence of conflicting visual information, our judgments of sound can be distorted, as demonstrated by the McGurk effect (McGurk and MacDonald, 1976). A few studies have tried to determine what causes differences in individual susceptibility to McGurk effects and what factors can affect AV fusion.

Proverbio et al. (2016) wanted to find out whether musical training would allow people to resist the McGurk effect, using a similar design (A, V, and AV). Like trans listeners in our study, musicians were able to ignore visual cues and focus on sound, resulting in AV ratings that were closer to A ratings. This suggests that training in auditory skills can help people evaluate sound more objectively. Thus, it would be interesting to determine whether our main finding (trans resistance to AV integration) would be reduced among musicians and amplified among non-musicians. Our study did not track musicianship but, given the relatively large samples used here, it is rather unlikely that the effect would be entirely due to TRANS participants being somehow more musically trained than the CIS participants recruited here.

Chládková et al. (2021) tested the way young and older adults responded to the McGurk effect at the very beginning of the mask-wearing mandates in Czechia and a month later to see whether speech perception relied less on visual cues after being deprived of lip/articulatory motion. Participants were presented with congruent AV stimuli (which served to exclude unreliable participants) and McGurk stimuli. The same experiment was repeated a month later but, unfortunately, not all participants in the second experiment were the same as in the first. A longitudinal design was chosen for 41 (young) students while a cross-sectional design was chosen for adults covering a larger age range. Bearing these methodological differences in mind, results showed that young people reduced their reliance on visual cues, whereas older people increased their reliance after a month of using face masks. Women exhibited a larger McGurk effect than men in both sessions (before and after a month of mask wearing), and they also relied less on auditory cues after a month of mask wearing while men relied more on them. This puzzling result shows that the relationship between different modalities is more complicated than what could be speculated based on the quality and availability of environmental cues. There are additional socio-psychological factors at play and the current study certainly adds support to the idea that the weighing of audiovisual cues is malleable.

Brown et al. (2018) attempted to uncover the cognitive and perceptual reasons why the McGurk effect might or might not occur. They investigated the effects of lipreading ability, response to ambiguous sounds (continuum between [s] and [ʃ]), level of cognitive ability (processing speed and working memory), and attention. They found that only lipreading skill predicted susceptibility to the McGurk effect. This sort of enterprise is incredibly useful to our present goal. If we are to develop behavioral training methods to counteract harmful voice appraisals, we need to focus on the few skills that can be sharpened (quickly) to resist AV integration. Brown et al. suggest that we turn our attention to the quality of visual cues and the efficiency with which they are extracted. However, this is rather at odds with the previous studies: Proverbio et al. (2016) suggested sharper auditory skills would be beneficial, and Chládková et al. (2021) pointed toward higher-level cognitive processes including learning. This leads us to speculate that the strength of AV integration might depend more on the goal of the task and peculiarities of the experimental design, rather than being a stable trait that can only be changed slowly and through much exposure. This calls for replications of Brown et al.’s study in the context of speaker’s and singer’s voice appraisal.

In the same vein, Nahorna et al. (2012) sought ways to stop or limit fusion of AV materials by “priming” participants to process AV stimuli in a coherent (multimodal) manner or with dissociated modalities. They preceded a test McGurk stimulus by either a congruent or an incongruent AV sample. They found that an incoherent sample (consisting of 5 syllables dubbed over the speaker saying a sentence) could greatly reduce the McGurk effect. This is a remarkable proof-of-concept that it is possible to prevent AV fusion simply by facilitating or hindering participants’ multisensory processing briefly before a test (another indication of the task dependency of this resistance). But this demonstration lacks a good deal of ecological validity. When a speaker or singer auditions for a role, there is little one could do in practice to force the judges’ brain to question the synchronization or congruency of A and V materials. So, unfortunately, we would need to find interventions that lead to more permanent changes in modality weighting or in voice-body expectations, not just a priming effect that carries over for a few seconds.

Finally, in terms of brain activity, we know that visual input is integrated into auditory processing in the periphery of the auditory cortex (Atilgan et al., 2018). Using fMRI, Nath and Beauchamp (2012) found a stronger response in the superior temporal sulcus (STS) on the left side when a person is more likely to perceive a McGurk effect. In the light of the present findings, the group difference in susceptibility to body/face biases is presumably substantiated by neurological markers, and therefore we would imagine trans participants to exhibit a weaker response in the STS than cis participants in an experimental design similar to ours. However, the lateralization of STS activity suggests that AV integration may differ considerably in the context of language versus musical or social contexts, whereas behaviorally this does not seem to matter (i.e., mode had negligible role on the AV shifts).

### 5.2. Trans individuals: from vocal hardship to expertise in voice

Most previous studies on transgender people and voice have focused on voice dissatisfaction in the trans community (McNeill et al., 2008; Hays, 2013; Valente and de Medeiros, 2020; Menezes et al., 2022), acoustic parameters thought to be associated with gender perception from the cisgender listener’s perspective (Coleman, 1983; Gelfer and Schofield, 2000; Gelfer and Bennett, 2013; Dahl and Mahler, 2020), and trans linguistics (Gratton, 2016; Zimman, 2020). The present study turned the tables and showed that, when involved in the tasks as voice judges (rather than when their involvement is limited to voicing stimuli), trans individuals can leverage their life experience with voice and identity. *There is, to our knowledge, no study that ever demonstrated a trans listener’s advantage in voice evaluation*. These findings teach us that it is possible to learn, perhaps through extensive training and exposure, to resist preconceived and culturally ingrained voice-body expectations when evaluating voices.

Meanwhile, a few studies exist, with trans individuals as actors, which aimed to assess how trans voice may be unfairly perceived. This is problematic as it excludes transgender people from their own care and assumes that achieving a “cis-passing” voice is always the ultimate goal of trans speakers and singers. Furthermore, it perpetuates traditional binary ideals of femininity and masculinity, which is damaging to all voice users (not just trans ones), when gender theory in general has moved away from the binary and increasingly toward the spectrum (Butler, 1990; Halberstam, 1998/2018; Williams, 2015; Morgenroth and Ryan, 2018). This spectrum should then be present in both vocal production and perception, as in other areas of gender expression.

Perhaps closest to the current paradigm, Van Borsel et al. (2001) examined the interaction of physical appearance and voice in gender perception of trans women. They divided listeners into two balanced groups: one that would first evaluate V-only stimuli and then AV stimuli and the other group who would evaluate A-only stimuli followed by AV stimuli. This separation of participants into two groups was far from ideal but was done because of concerns that participants would recognize the actors in the different modes (exacerbated by the fact that few actors/stimuli were used). Responses were provided to assess gender instead of voice type, but at least they varied on a continuous scale. For 9 out of 14 trans women, participants provided higher ratings (more feminine) in V-only, intermediate in AV, with lowest ratings for A-only. This is the pattern the authors expected, on the basis that trans women voices would convey too many masculine features. But this did not happen in the five other speakers, for which participants actually provided higher ratings in A-only than in AV and V-only (suggesting that participants were then perhaps judging the visual stimuli unfairly). Contrary to the authors’ beliefs, we think the key point is that while there is systemic prejudice against trans people in society, prejudicial judgments are not necessarily made systematically. We must not generalize about individual trans people or about their vocal treatment plans; but rather we should consider the specific needs and challenges of each person. What Van Borsel et al.’s (2001) findings do prove to be systematic is the response to AV stimuli, which is always in between that of A-only and V-only stimuli. In other words, this is yet another demonstration that AV integration is hard to resist, and this may perhaps be particularly true when assessing trans women’s voices.

In a similar study of trans men’s voices, Van Borsel et al. (2009) found little bias (in either direction, although this is difficult to assess given that participants were again split in two groups evaluating A to AV in one group, and V to AV in the other), with perhaps a small dependency on the speaker’s habitual voice pitch. Comparing their findings across the two studies, the authors argued that high voices in men are more accepted than low voices in women. To our knowledge, no study has ever demonstrated this to be true, but it has been written about since then (Block et al., 2018) and it is anecdotally accepted within the trans community that trans men attract less attention in general than trans women (Atkinson, 2018; Kiss, 2018; Alter, 2022). These anecdotal observations point to a major area of concern in women and gender studies, and we did find evidence in our study that those whose appearances were more masculine than their voices suggested suffered less (see 4.5). From this, we can infer that trans women with voices perceived as more masculine that their appearance suggests generally experience more bias from society than trans men whose voices may be higher or brighter than their appearance suggests. More generally, people with a feminine appearance (whether cis or trans) may be more closely scrutinized than those with a masculine appearance.

Of note, the two Van Borsel et al. are problematic due to (1) outdated language and ideas about sex and gender and (2) the challenges associated with properly examining a pattern that is inherently bimodal. However, one finding that drew our attention is the fact that the listeners’ group (whether they were lay people or speech-language pathologists, SLPs) had no role. Assuming that the SLPs had some level of professional or at least academic expertise in voice relative to lay people, this finding might seem in contradiction with the present ones. Nonetheless, it reinforces the notion that the trans listener advantage observed here comes from a first-hand experience with body-voice dissociation, and not just from greater knowledge of the voice apparatus. The current study uses a somewhat similar method of evaluating speakers with the advantage of capturing within-subject judgments in A-only, V-only, and AV stimuli, with a much larger sample size of listeners, a greater diversity of actors, still placing gender implicitly on a continuum. On this basis, we believe that the impact of physical appearance in voice evaluation is strongly dependent on the listener’s characteristics (contrary to their conclusion), but perhaps not necessarily their knowledge of voice/speech disorders.

AV integration set aside, there are a few studies that failed to show a trans listener advantage. For example, interested in how a trans voice might be perceived over the course of hormone replacement therapy (HRT), Brown et al. (2020) showed that cis listeners just like trans listeners categorized a trans masculine speaker as “definitely female” until week 14 of HRT and shifted their response to “definitely male” at week 28. Other than demonstrating that HRT is a successful approach to changing voice characteristics in trans masculine speakers, their lack of group difference suggested that trans listeners follow the same gender “rules” as cis listeners when judging A-only stimuli. This is not inconsistent with the present data as gender had negligible effect in A-only conditions (TRANS listeners tended to use a larger scale overall, not a different boundary between the three traditionally male and the three traditionally female categories). The novelty that our study provides is that trans listeners would be better at relying on these auditory cues alone and ignoring visual cues which hinder a more subtle categorization. In other words, we would suspect that the lack of group difference observed in Brown et al. (2020) would not necessarily hold with AV materials and more ecological settings.

Hope and Lilley (2022) borrowed the framework of language acquisition to theorize that gender perception (e.g., just like vowel perception) would differ depending on whether actors fell in “native” compared to “non-native” regions of their (abstract) gender space. With the help of artificial intelligence, they generated synthetic nonbinary voices with a neutral F0 but within traditional regions for contour and vowel formants (masculine, feminine) or more untapped regions of contour and vowel formants (neutral, mixed). Cis and gender-expansive listeners evaluated voices both along a continuum from male to female and categorically by checking one of five boxes: man, woman, nonbinary, agender, and genderfluid. Results showed that cisgender people tended to rate voices on the extremes of the continuum, while the gender expansive listeners placed voices more in the center of the spectrum. The more extreme ratings by cis listeners could be a consequence of greater dichotomy compared to gender expansive listeners who were happy placing speakers in the middle. This pattern seems largely consistent with our message that trans listeners are better (more nuanced) judges of voices, possibly even in the absence of visual cues.

Other researchers have looked at the role of sexual orientation, not gender identity, on voice perception of cis and trans speakers with A-only stimuli (Hancock and Pool, 2017). In a first task, listeners rated speakers’ gender along a continuum just like the current study, and there was no significant interaction between the listeners’ sexual orientation and the speakers’ gender. Despite the authors’ claim (from non-significant results), this suggests that the listener’s sexual orientation is rather irrelevant to perception of cis or trans speakers. In a second task, listeners were asked to follow up with their continuous rating and make a dichotomous categorization between male and female. Once again, there was no interaction between the listener’s sexual orientation and the speakers’ gender. Also, note that in both tasks, it made no difference whether the listeners were of male or female sex, and no difference whether they had any contact with the LGBTQ community. Hancock & Pool’s findings indicate that sexual orientation is likely to play a negligible role in the evaluation of A-only stimuli, but it is not clear whether these results would hold with AV materials. We believe that trans people, of any sexual orientation, would have more cause to focus on gender-loaded cues in voices than straight or non-straight cisgender people.

Finally, Owen and Hancock (2010) compared trans women’s self-perception of voice with cis listener perceptions and found that both groups were in agreement. They concluded that trans women’s self-ratings were therefore useful for monitoring and evaluating their own progress in voice therapy. This study, although perhaps well-intentioned, placed cis listeners opinions as the ground truth with the obtention of “cis-passing” voices as the goal. Inadvertently this invalidates the independent opinions of transgender people and again reduces agency in their own care. Moreover, it is a misconception that transgender people, in transitioning, are always aiming for a cis-passing appearance or voice. Besides being a sweeping generalization about a diverse umbrella group, this assumption erases trans culture in which ideas about femininity, masculinity, and other genders exist, but are not necessarily the same as in cis culture. While the goal of a trans person may indeed be to pass as their gender, this does not require passing as cis. If the trans advantage observed in our study is replicated in other studies and confirmed by several lines of investigation, we may eventually consider trans judges’ opinions of voice to be the gold standard in the future.

### 5.3. The role of mode and emotional expressivity in gender perception

Finally, it should be noted that several parameters have been found to influence gender perception in voice. Pitch (F0), resonances of the vocal tract (shape of spectral envelope and vowel formant placement) and articulation (how we pronounce words using the lips, teeth, tongue, palate, glottis, nasal cavity, pharynx) all affect vocal timbre and are perhaps the most salient properties related to gender perception. Other factors play a role as well: melodic contour, word stress and word choice, and non-verbal cues such as posture and eye contact (Jackson Hearns, 2018). Many of these influences strongly apply to speech but break down in singing because the vocalist has limited interpretive freedom in musical contexts. In this study, we found singing to induce a more feminine percept and this could be utilized by trans individuals in either direction (exaggerating contours and emotional expressivity in speech to sound more feminine or singing in a flatter manner more speech-like to sound more masculine). These techniques could be used by trans individuals as speakers to influence how they are perceived in the world, but they will not address the problem at hand, which concerns listener prejudice.

## 6. Conclusion

Although it may seem like the sound of a voice should be the primary factor, perhaps the only factor, considered when listeners perceive and classify a voice, in reality due to AV integration, a speaker’s or singer’s appearance affects how listeners categorize and evaluate that voice. This has led to the exclusion of transgender singers (and, to some extent, cisgender singers) who do not meet voice-body expectations from formal singing experiences, such as participation in community or professional choirs. It also affects the classification of professional opera singers, which can be detrimental to their long-term vocal health. This study found that transgender listeners, on average, were better suited than cisgender listeners to objectively identify a singer’s or speaker’s voice type, perhaps because they were better able to ignore the actors’ appearance, a finding that opens exciting avenues to combat implicit (or sometimes explicit) biases in voice evaluation.

This work also has implications for the field of trans voice, showing that cisgender listeners are more pulled toward a binary gender perception when visual information is available, which could either help or hinder a trans person depending on their visible gender expression. Perceived voice-body mismatches not only affect singers but, worse yet, can lead to obstacles in the daily lives of transgender people who must use their voices (and thus risk outing themselves) to secure medical services, take public transportation, order food, etc. It is therefore crucial that we study the conditions under which visual biases in voice evaluation occur so that we might begin to break down both the explicit and implicit biases that exist in gender perception of the voice.

## Data availability statement

The datasets presented in this study can be found in online repositories. The name of the repository and accession number can be found at: Open Science Framework (OSF), https://osf.io/346zj.

## Ethics statement

This study, which involved human participants, was reviewed and approved by the Research Ethics Unit (Office of Research) at Concordia University, ref.: 30014709. The participants provided their online consent (on Pavlovia) to participate in the study.

## Author contributions

JMK and MD conceived and designed the experiment, and selected the stimuli within the RAVDESS database. MD and AS coded the experimental interface. JMK and MD analyzed the data and worked on figures with the help of AS. All authors contributed to the article and approved the submitted version.

## Funding

This research was supported by internal funds (Concordia Univ.) to MD and a Frederick Lowy Fellowship (Concordia University) to JMK.

## Conflict of interest

The authors declare that the research was conducted in the absence of any commercial or financial relationships that could be construed as a potential conflict of interest.

## Publisher’s note

All claims expressed in this article are solely those of the authors and do not necessarily represent those of their affiliated organizations, or those of the publisher, the editors and the reviewers. Any product that may be evaluated in this article, or claim that may be made by its manufacturer, is not guaranteed or endorsed by the publisher.

## Supplementary material

The Supplementary material for this article can be found online at: https://www.frontiersin.org/articles/10.3389/fpsyg.2023.1046672/full#supplementary-material

## Footnotes

^1^See https://zenodo.org/record/1188976#.ZEpP9uzML0o.
