# Supplementary material for: Visual biases in evaluation of speakers’ and singers’ voice type by cis and trans listeners
Source: Front Psychol. 2023 May 2;14:1046672. doi: 10.3389/fpsyg.2023.1046672 (PMC10187036; doi:10.3389/fpsyg.2023.1046672)
Supplement: Supplementary file 1 [file Data_Sheet_1.pdf]

Supplementary Materials 1: no role for emotional or semantic content

Our material consisted of sentences enacted with a neutral or an emotional manner. Besides, emotional stimuli were chosen to be as vibrant as possible by selecting only the recordings where actors were instructed to act emotions intensely (there were actually two levels of emotional intensity in the original materials developed by Livingstone and Russo, 2018). Here we replicated our repeated-measures ANOVA with two within-subject factors (voice category, emotional intensity) and one between-subject factor (group) to demonstrate that our findings disregarded this factor.

As illustrated in the top panel of Fig. A1, there was no main effect of emotional intensity [$F(1,164)=1.6$, $p=0.205$, $\eta^2<0.001$] and its interaction with category missed significance [$F(4.3,697.9)=2.3$, $p=0.056$, $\eta^2=0.003$]. Its interaction with gender [$F(1,164)=0.7$, $p=0.413$, $\eta^2<0.001$] or in a 3-way [$F(4.3,697.9)=1.7$, $p=0.138$, $\eta^2=0.003$] were not significant either. Thus, emotional intensity did not impact our earlier analysis. Note that emotional stimuli tended to lead to higher/brighter ratings than neutral stimuli, bearing some resemblance to the mode effect discussed in the article (section 4.2), where expressive voices and faces could elicit a sense of enthusiasm that might conceivably be perceived as lighter, more joyful, and perhaps more feminine. However, since it applied to both modalities (despite some differences across categories) and roughly equally to both groups, it had negligible influence on the magnitude of the AV shift, our key finding.

Semantically, the entire material was composed of two sentences: “dogs are sitting by the door” and “kids are talking by the door”. We did not expect the choice of words to have any impact on our results, but we examined this factor here in the same vein as mode or emotional intensity. As

illustrated in the bottom panel of Fig. A1, there was no main effect of sentence [$F(1,164)=0.2$, $p=0.635$, $\eta^2<0.001$], and sentence did not interact with gender [$F(1,164)=1.2$, $p=0.273$, $\eta^2<0.001$], with actor category [$F(5,820)=0.1$, $p=0.988$, $\eta^2<0.001$], or in a 3-way [$F(5,820)=1.1$, $p=0.363$, $\eta^2=0.001$]. Therefore, the semantic content of the stimuli had no influence in our key finding.

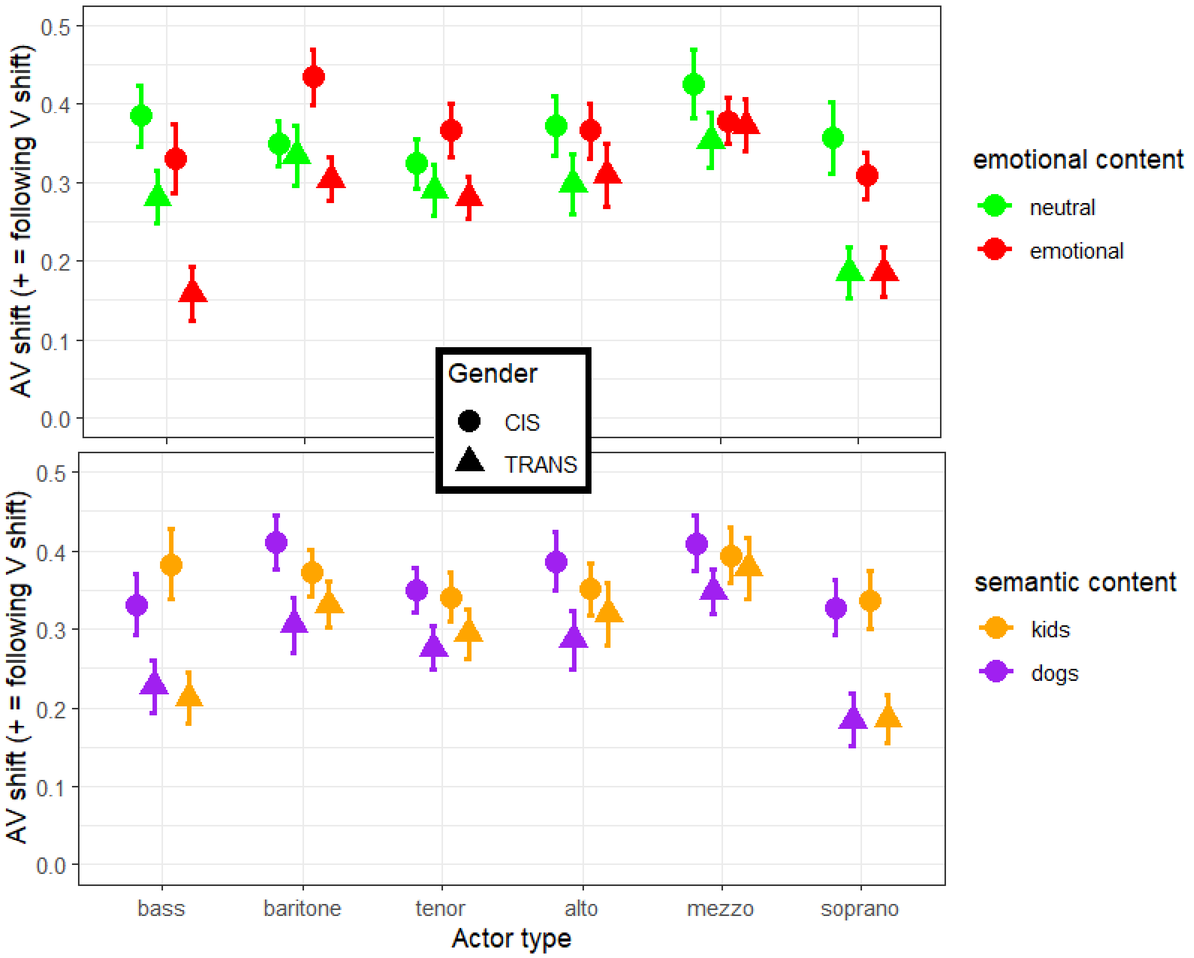

Fig A1: same as bottom panel of Fig.5 but replacing mode by emotional content (top) or by semantic content (bottom).
