# Supplementary material for: Visual biases in evaluation of speakers’ and singers’ voice type by cis and trans listeners
Source: Front Psychol. 2023 May 2;14:1046672. doi: 10.3389/fpsyg.2023.1046672 (PMC10187036; doi:10.3389/fpsyg.2023.1046672)
Supplement: Supplementary file 2 [file Data_Sheet_2.docx]

**Supplementary Materials 2: no distinction between binary and non-binary within the TRANS group**

The 81 individuals that formed the TRANS group were able, to some degree, to resist the visual biases induced by the actors’ physical appearance. This group, however, was heterogeneous and composed of 26 binary and 55 non-binary adults. In this additional analysis, we questioned whether this sub-distinction could have revealed different patterns of AV-shift than those reported in the article.

As illustrated in Fig. A2, there was a main effect of actor category [F(4.4,344.1)=7.5, p<0.001, η^2^=0.030], confirming that the bias was reduced for basses and sopranos (mentioned in the article). But there was no main effect of subgroup [F(1,79)=2.1, p=0.152, η^2^=0.010], and subgroup did not interact with category [F(4.4,344.1)=0.6, p=0.664, η^2^=0.002], with mode [F(1,79)=1.0, p=0.325, η^2^<0.001], or in a 3-way [F(4.5,353.8)=0.6, p=0.663, η^2^=0.002]. In other words, this analysis did not provide any further understanding of the data: TRANS individuals behaved rather homogeneously with respect to these biases, whether they were binary or non-binary.


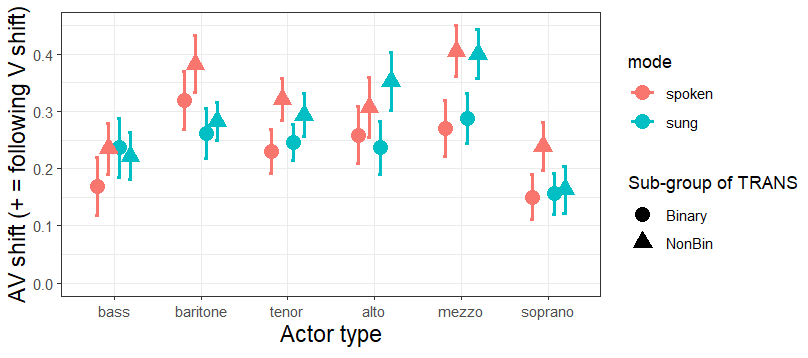
Fig A2: same as bottom panel of Fig.5 but splitting the TRANS population into two sub-groups: binary vs non-binary individuals (regardless of their assigned sex at birth).
