# Supplementary material for: Visual biases in evaluation of speakers’ and singers’ voice type by cis and trans listeners
Source: Front Psychol. 2023 May 2;14:1046672. doi: 10.3389/fpsyg.2023.1046672 (PMC10187036; doi:10.3389/fpsyg.2023.1046672)
Supplement: Supplementary file 3 [file Data_Sheet_3.docx]

**Supplementary Materials 3: a small role for assigned sex**

In contrast to our conclusion (pointing to cognitive and malleable influence of visual cues in voice appraisal), certain authors have argued for biological, and in fact evolutionary, factors of gender perception (Smith, Grabowecky, & Suzuki, 2007). In this additional analysis, we questioned whether assigned sex at birth (90 female and 76 male, regardless of their gender) could have revealed a different insight into the patterns aforementioned.

As illustrated in Fig. A3, the main effect of sex was not significant [F(1,164)=3.2, p=0.077, η^2^=0.008], but it interacted with actors’ category [F(4.4,726.7)=3.0, p=0.014]. Pairwise comparisons revealed that participants assigned male tended to resist biases compared to participants assigned female, for baritones (p=0.013), altos (p=0.038), and mezzos (p=0.038), by 0.09 point in each case, but not for basses (p=0.334), tenors (p=0.069), or sopranos (p=0.355). Interestingly however, the difference headed in the opposite direction for sopranos (participants assigned female exhibiting less bias than participants assigned male). Also, sex did not interact with mode [F(1,164)=1.0, p=0.327, η^2^<0.001] or in a 3-way [F(4.6,754.6)=1.1, p=0.376, η^2^=0.001]. To summarize, there might be a (small) role for sex in our study, but it was not systematic across actors. We have little speculation to offer for this phenomenon. Following the same reasoning as our interpretation of the TRANS advantage, one could imagine participants having more ease to dissociate appearance from voice within their own sex precisely because they would have more implicit knowledge about it (remember that this analysis includes both CIS and TRANS participants). This points again to the role of first-hand life experience with subtle mismatches for example during puberty. If this were the case, one could expect a greater bias for opposite-sex actors than same-sex actors. The reverse direction of the effect of sex for sopranos offer tantalizing clue that this might happen, but for some reason the asymmetry was not systematic.


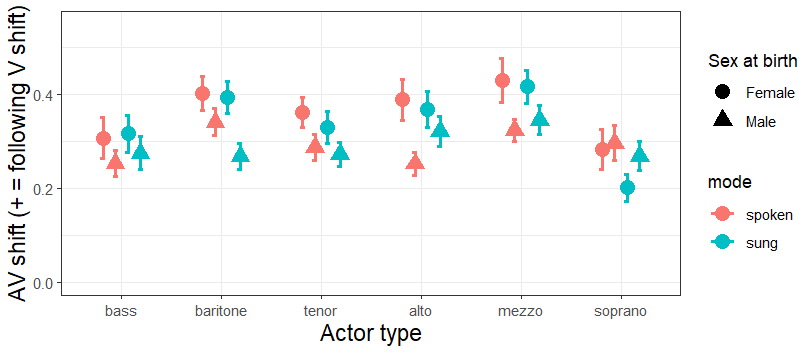


Fig A3: same as bottom panel of Fig.5 but reallocating each individual based on their assigned sex at birth, rather than based on their gender.
