# Supplementary material for: Visual biases in evaluation of speakers’ and singers’ voice type by cis and trans listeners
Source: Front Psychol. 2023 May 2;14:1046672. doi: 10.3389/fpsyg.2023.1046672 (PMC10187036; doi:10.3389/fpsyg.2023.1046672)
Supplement: Supplementary file 4 [file Data_Sheet_4.pdf]

Supplementary Materials 4: Bootstrapping

Most of the analyses presented in the article used traditional (frequentist) statistics. Typically, the analysis of the data of Figure 4 (section 4.2) and Figure 5 (section 4.3) used repeated-measures ANOVA with one between-subject factor (group, CIS vs TRANS) and two within-subject factors (voice category with 6 levels, and mode: spoken vs sung). These analyses rely on a number of assumptions (independence of the observations, normality of the DV, homogeneity of variance between the two populations, sphericity across the 6 voice categories). These assumptions were not always met: typically, sphericity was often violated for the effect of category or its interaction with other factors, and homogeneity of variance between CIS and TRANS participants was not always respected in each category × mode condition. Thus, one could question the credibility of p-values reported. To address this concern, here, we ran 5000 iterations of a permutation technique (shuffling the DV across the whole dataset without replacement) to estimate the distribution of each main effect and interaction under the null hypothesis and generate a p-value of how extreme the real F values happened to be. They are reported in this table, and were all in close agreement with the p-values derived from theoretical F distributions reported in the main article.

	A-only	V-only	AVshift (signed)
Gender	p=0.380	p=0.799	p=0.006
Category	p<0.001	p<0.001	p<0.001
Mode	p<0.001	p<0.001	p=0.278
Gender × Category	p=0.818	p=0.986	p=0.053
Gender × Mode	p=0.095	p=0.315	p=0.668
Category × Mode	p<0.001	p<0.001	p=0.181
Gender × Category × Mode	p=0.767	p=0.855	p=0.600

Below are additional figures showing the actual distribution of the 5000 F-values from resampled data in each main effect and interaction, along with a vertical red line showing the cutoff at 95% of the distribution.

A-only:

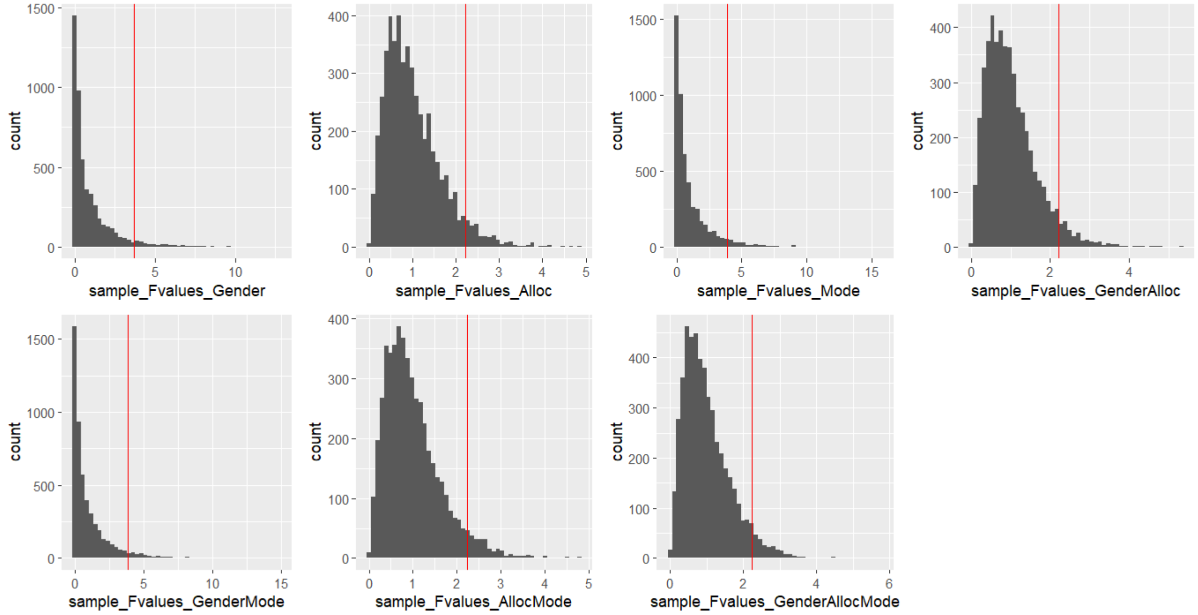

V-only:

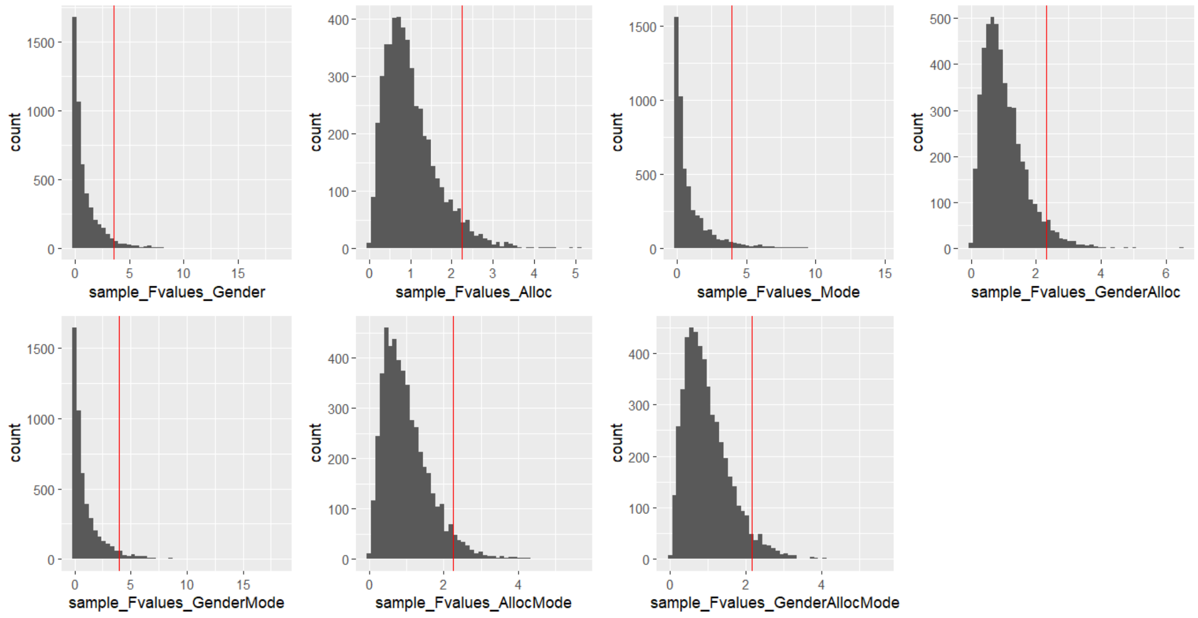

AV-shift:

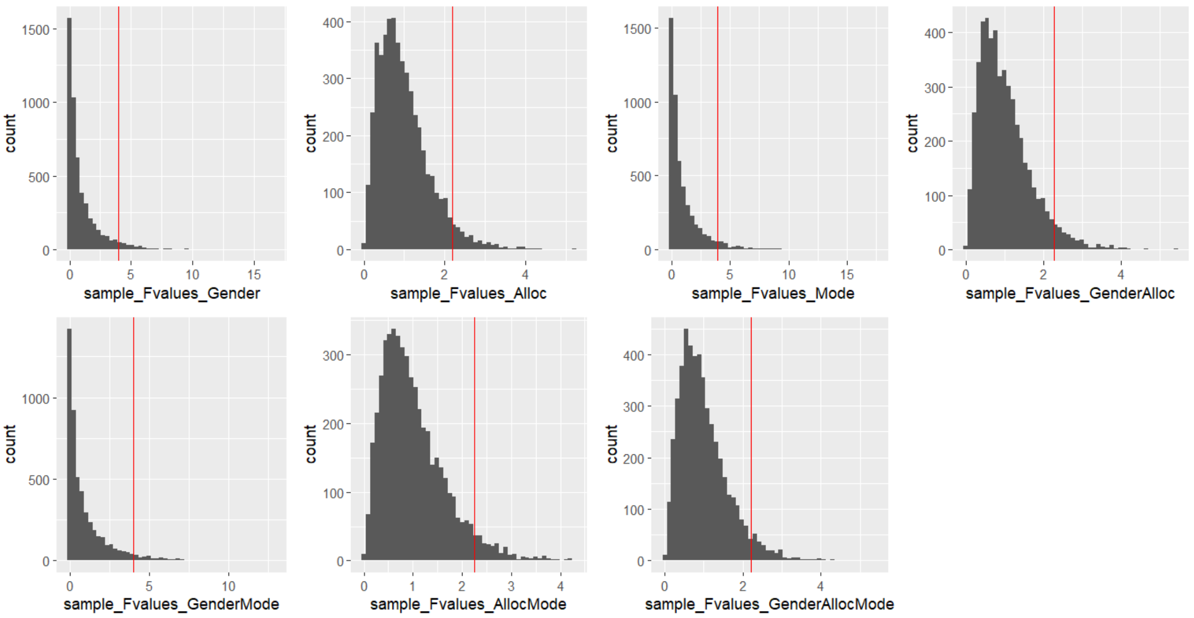
count
sample_Fvalues_Gender
sample_Fvalues_Alloc
sample_Fvalues_Mode
sample_Fvalues_GenderAlloc
sample_Fvalues_GenderMode
sample_Fvalues_AllocMode
sample_Fvalues_GenderAllocMode
