# Supplementary material for: Visual biases in evaluation of speakers’ and singers’ voice type by cis and trans listeners
Source: Front Psychol. 2023 May 2;14:1046672. doi: 10.3389/fpsyg.2023.1046672 (PMC10187036; doi:10.3389/fpsyg.2023.1046672)
Supplement: Supplementary file 5 [file Data_Sheet_5.pdf]

Supplemental Materials 5: Gender Views Questions 1-20 (open source from the *Commonwealth of Learning*)

1. The birth of a boy in more important than that of a girl.
2. Sex and gender are the same thing.
3. A girl’s appearance matters more than a boy’s.
4. It is women’s responsibility to care for children.
5. Boys are often better in mathematics and science than girls are.
6. It is probably a more worthwhile investment to spend money on sending boys to school than girls.
7. Men and boys attain higher literacy levels than women and girls.
8. Girls are less able than boys to withstand the physical demands of contact sports such as football, rugby or martial arts.
9. Men are more competent owners of property and land than women.
10. Men tend to be more reliable than women about paying back borrowed money.
11. If women take maternity leave, they should not complain if their careers stall.
12. These days, women and men with similar qualifications earn equal pay.
13. As women’s rights have increased, men seem to have increasingly less status and position in society.
14. Women cannot work and take care of their families at the same time.
15. Men tend to have greater responsibilities than women.
16. A woman should not put her career before her husband’s.
17. Women cannot succeed in male-dominated professions such as engineering.
18. When making decisions, men tend to be more rational than women.
19. Women are too emotional to be effective leaders.
20. Women and men running executive boards together causes problems.
